# Ethanol sclerotherapy versus cystectomy in the management of endometriomata: a systematic review and meta-analysis

**DOI:** 10.1186/s12905-026-04645-2

**Published:** 2026-07-18

**Authors:** Sarah L. M. Bennet, Sangeetha Nagenthiran, Thomas Bainton, Jeffery Ahmed, Victoria Male, Mark Johnson

**Affiliations:** 1 Department of Obstetrics and Gynaecology, Chelsea and Westminster Hospital NHS Trust, London, SW10 9NH UK; 2 Department of Metabolism, Digestion and Reproduction, Imperial College London, London, SW10 9NH UK

**Keywords:** Endometrioma, Sclerotherapy, Ovarian Reserve, Fertility, Endometriosis

## Abstract

**Background:**

The management of endometriomata remains a challenge, with current options associated with either high recurrence rates or significant reduction in ovarian reserve. Ethanol sclerotherapy has been suggested as a treatment that is effective while maintaining ovarian reserve.

**Methods:**

PubMed, EMBASE, MEDLINE, SCOPUS and CENTRAL were searched with no restriction on date or language. Keywords and MESH terms for ‘endometrioma’ AND ‘ethanol sclerotherapy’ AND ‘cystectomy’ were used. Studies were included if they made a direct comparison between ethanol sclerotherapy and cystectomy in the management of endometriomata in women of reproductive age. Randomised controlled trials (RCTs), cohort or case control studies were included. Searches were performed 11th February 2025 and repeated 21st August 2025. Risk of bias was assessed using ROB2 for RCTs and ROBINS-I for observational studies.

**Results:**

Searches identified 172 studies with 16 of these fitting the inclusion criteria.

Unfortunately, a formal analysis of between group change in Anti-Mullerian hormone (AMH) was not possible due to insufficient reporting of change score variance data, as such an exploratory between group pre- and post- analysis was performed instead. This found no significant difference in AMH at baseline (mean difference (MD) 0.02, 95% CI –0.20 to 0.25, *p* = 0.830) while post procedure AMH was significantly higher in the sclerotherapy arm (MD 0.75, 95% CI 0.43 to 1.06, *p* < 0.001). Overall risk of bias was high; however, similar results were seen following a sensitivity analysis excluding studies at highest risk of bias (high for ROB2 and critical for ROBINS-I) (MD -0.25, 95% CI -0.57 to 0.08, *p* = 0.136 and MD 0.60, 95% CI 0.22 to 0.97, *p* = 0.002, respectively). Studies reporting on Antral Follicle Count (AFC) gave conflicting results and all were assessed as at high or critical risk of bias. No significant difference was seen in recurrence (LogOR 0.28, 95% CI –0.57 to 1.12, *p* = 0.523). Rates of serious complications were low at 0.24% for sclerotherapy and 0.76% for cystectomy. It was not possible to pool data on symptomatology. Number of oocytes retrieved was higher in the sclerotherapy arm (MD 3.23, 95% CI 1.34 to 5.12, *p* < 0.001). Number of mature oocytes retrieved was also higher in the sclerotherapy arm (MD 2.79, 95% CI 1.08 to 4.51, *p* = 0.001), however this became non-significant when studies at the highest risk of bias were excluded (MD 0.79, 95% CI -0.61–2.19,* p* = 0.27). Data on pregnancy rate could not be pooled due to the wide variation in definition of clinical pregnancy. No significant difference was seen in live birth rate between interventions (logOR 0.59, 95% CI -0.30 to 1.48, *p* = 0.192).

**Conclusions:**

Our results show ethanol sclerotherapy maintains ovarian reserve when compared to ovarian cystectomy in the treatment of endometrioma, as evidenced by the higher post-procedure AMH levels and increased number of retrieved oocytes at IVF seen following sclerotherapy. However, due to the high risk of bias and flaws in design of included studies, high-quality randomised controlled trials with unified outcome standards are urgently required to confirm these findings and verify the protective benefit of ethanol sclerotherapy. This would support patients and their clinicians in decisions regarding management of endometriomata, particularly when fertility preservation is required.

**Trial registration:**

This review was prospectively registered with the International Prospective Register of Systematic Reviews (PROSPERO). The registration number is CRD42024625920.

**Supplementary Information:**

The online version contains supplementary material available at https://doi.org/10.1186/s12905-026-04645-2.

## Background

Endometriosis is a chronic disease, estimated to affect up to 11% of women of reproductive age [1, 2]. It is characterised by the presence of endometrial tissue outside the uterine cavity, most commonly within the pelvis [3]. Pelvic endometriosis typically presents with symptoms of dysmenorrhoea, dyspareunia and chronic pelvic pain [4, 5]; however, symptoms are varied and may include bowel symptoms, urinary symptoms, nausea, fatigue or it may be asymptomatic [2, 6]. Endometriosis may also present with subfertility, being diagnosed in up to 50% of women undergoing fertility investigation [7]. These symptoms can have a significant impact on quality of life and a high socioeconomic burden, similar to chronic diseases such as Diabetes Mellitus and Crohn’s Disease [4, 8].

Endometriosis is often categorised into peritoneal, ovarian and deep infiltrating endometriosis (DIE) [9], however, the severity of disease seen on imaging or at surgery has little correlation with patient symptoms [10]. The only association consistently identified is dyspareunia with lesions in the posterior cul-de-sac [11, 12]. The prevalence of endometriomata is 20–53% of women with endometriosis [13, 14]. They are rarely seen independently [13, 14] and may be an indication of more severe endometriosis when seen with DIE [15].

Medical management of endometriosis involves analgesics and hormonal therapies such as combined contraceptives, progestogens, gonadotrophin releasing hormone (GnRH) agonists or antagonists, or aromatase inhibitors [16]. A number of these may lead to a reduction in endometrioma size [17], although the clinical significance of this reduction is debated, and there is a suggestion some may preserve Anti-Mullerian Hormone (AMH) levels [18]. Unfortunately, none of them are suitable for women who are actively trying to conceive due to their contraceptive action.

Surgical treatment of endometriomata may be considered due to pain symptoms in cases where medical treatment is unsuccessful or inappropriate, to facilitate assisted reproductive technology (ART) or to improve the chances of natural conception [16]. While there are a number of surgical techniques available, they all have their limitations.

Incision and drainage is the least invasive surgical option, however, it is associated with recurrence rates of 80–84% at 6 months [19, 20]. This can be improved by combining cyst drainage with an ablation technique [19], such as laser, plasma energy or electrocoagulation. Unfortunately, even with ablation the recurrence rates remain higher than following cystectomy [19, 21] and, importantly, cystectomy also shows greater symptom improvement [21]. Cystectomy has long been considered the gold standard for treating endometriomata, but despite this there are concerns regarding the impact cystectomy might have on a woman’s ovarian reserve, with an AMH reduction of 30–39.5% seen with unilateral endometrioma and 44–57% with bilateral endometriomata [22, 23]. Even more concerning is the post-surgical ovarian failure rate following excision of bilateral endometriomata that has been reported as 2.4% [24]. Of note, drainage and ablation of endometriomata can also lead to a reduction in ovarian reserve [25], with electrocoagulation showing a similar decline to that of cystectomy [26].

With the significant impact endometriosis may have on a woman’s quality of life [4, 8] and the fact that it is a disease affecting young women who may have future fertility desires, it is important to identify management options that provide good symptom control, low recurrence rates and that preserve ovarian function to maximise fertility.

Ethanol sclerotherapy is an increasingly used technique that has shown some promise in addressing these issues. It involves drainage of the endometriomata and chemical ablation of the cyst wall with a concentrated ethanol solution [27, 28]. Ethanol sclerotherapy has been shown to improve pain symptoms and quality of life [29], with low complication rates [30–32] and has reported recurrence rates that are similar to those of cystectomy (9–20% [30, 32, 33] and 3–30% [21, 34] respectively). A number of studies have shown AMH to remain stable following ethanol sclerotherapy [35–38], although a recently published single-arm, prospective cohort study looking at laparoscopic sclerotherapy and excision of pelvic endometriosis noted a significant decrease in AMH which the authors describe as similar to other ablative methods [32].

The effect of sclerotherapy on pregnancy outcome is unclear. When compared to conservative treatment in women undergoing in vitro fertilisation (IVF), ethanol sclerotherapy shows no difference in gonadotrophin dose required, number of oocytes retrieved, number of embryos or fertilisation rate [33, 39, 40], while live birth rate was found to be significantly higher following sclerotherapy in both studies that reported on it. This suggests the presence of an endometrioma may exert a negative effect on implantation or very early pregnancy, for example due to a pro-inflammatory environment, and that sclerotherapy might reduce this effect. This is supported by a previous study using donor eggs that found the presence of endometriosis negatively affects endometrial receptivity [41]. Conversely, studies comparing cystectomy to conservative management or drainage and ablation have shown no difference in clinical pregnancy rates or live birth rates between each technique [21, 42]. This would suggest that sclerotherapy might show improved pregnancy outcomes compared to cystectomy, however, a meta-analysis pooling three studies [31] found no significant difference in clinical pregnancy rates following IVF when sclerotherapy was compared to cystectomy.

Previous meta-analyses of ethanol sclerotherapy in the management of endometriomata did not limit the sclerosing agents assessed to ethanol [31, 43, 44], did not specify comparison between sclerotherapy and cystectomy for pooled data [31, 44, 45], had methodological errors evidenced by including studies that did not meet their search criteria [31, 43, 45], omitted widely available studies [46], were limited by number of studies [31], or did not analyse markers of ovarian reserve [43, 46]. Our objective was to perform a systematic review and meta-analyses of studies directly comparing ethanol sclerotherapy with the gold standard treatment of cystectomy, assessing markers of ovarian reserve, recurrence, complications, symptom relief and pregnancy outcomes.

## Methods

### Registration and protocol

This review was prospectively registered with the International Prospective Register Of Systematic Reviews (PROSPERO). The registration number is CRD42024625920. Amendments were made to clarify search strategy following advice from a librarian, to add an additional reviewer and to update outcomes measured to reflect available data.

### Eligibility criteria

Studies were included if they made a direct comparison between ethanol sclerotherapy, and cystectomy in the management of endometriomata in women of reproductive age. Sclerotherapy performed by both laparoscopic and ultrasound guided approaches were included. Laparoscopic sclerotherapy is performed under general anaesthesia with direct visualisation of the endometrioma. Ultrasound guided sclerotherapy is less invasive and can be performed as an outpatient procedure by either transabdominal or transvaginal approach depending on the cyst size. Studies were included if they reported on at least one of the described outcomes.

Studies were included if they were randomised controlled trials, cohort studies or case–control studies. Non-original research, case reports and case series were excluded. Non-English language studies were included in the search, however excluded from analysis if English translations could not be obtained.

### Information sources and search strategy

The systematic review and meta-analysis were performed as outlined by the Preferred Reporting Items for Systematic Reviews and Meta-analysis (PRISMA) guidelines. Searches were performed across EMBASE, PubMed, MEDLINE, SCOPUS and The Cochrane Library (CENTRAL) with no restriction on dates or language. No specific grey literature search was performed, however study authors were contacted to provide further information in cases where studies were registered, but not published, or for abstracts related to conference presentations. The initial search was performed 11th February 2025. A repeat search was performed 21 st August 2025. Searches were performed using keywords and MESH terms for endometrioma, ethanol sclerotherapy and cystectomy (see Additional File 1 for details of search strategy). Searches were performed for Endometrioma AND Sclerotherapy AND Cystectomy and all results screened.

### Study selection

All search results from each database were imported to the systematic review management tool Covidence. Two authors (SB, TB) independently reviewed titles and abstracts and discarded those that did not meet the inclusion criteria. Full text articles of the remaining studies were then independently assessed (SB, TB) and excluded if they did not meet inclusion criteria. Any disagreements were discussed with a third reviewer (SN) and a consensus reached.

### Outcomes and effect measures

The primary outcome to be analysed was change in markers of ovarian reserve from baseline to post-procedure as measured by AMH and antral follicle count (AFC). All timepoints described by authors were extracted.

Secondary outcomes included cyst recurrence, complication rates and symptomatology. Pregnancy and reproductive outcomes were also analysed, as measured by number of retrieved oocytes, number of mature oocytes, clinical pregnancy rate and live birth rate.

### Data collection process

Data were extracted using Covidence software. Data were extracted independently by two reviewers (SB, SN), any disagreements were discussed and a consensus reached. The plan was to extract data for the described outcome measures including mean difference in AMH, mean difference in AFC, recurrence rate, complication rate, symptomatology and pregnancy outcomes (number of oocytes retrieved, number of mature oocytes retrieved, clinical pregnancy rate and live birth rate).

Unfortunately, only one study [47] described mean change in AMH from baseline, with most studies reporting mean AMH before and after each intervention. As such, a formal analysis of between group change in AMH was not possible and an exploratory between group pre- and post- analysis was performed instead. A similar issue was encountered with AFC, which has been reported in a similar manner.

When available, data were extracted regarding study design, publication date, study funding, baseline characteristics of study participants and follow up period.

### Effect measures, data analysis and synthesis

Continuous data is described as mean and standard deviation, categorical data as numbers and proportions. Meta-analysis was performed for continuous data using a random effects model (Inverse-variance, restricted maximum likelihood method), with mean difference and 95% confidence intervals calculated. In cases where median and interquartile range were provided in the original data, these were converted to an estimate of mean and standard deviation using the formula provided by Wan et al. [48]. Where studies reported on multiple sclerotherapy interventions, results were combined to create a summary statistic for both groups [49]. For binary data, meta-analysis was performed using a random effects model (Inverse-variance, restricted maximum likelihood method), with log Odds Ratios and 95% confidence intervals. Where no events were seen in one or more arms a fixed correction of 0.5 was used. A p value ≤ 0.05 was considered statistically significant. Analysis was carried out using SPSS v30. Heterogeneity was assessed using I^2^.

Where meta-analysis was not appropriate results are synthesised descriptively.

All studies were initially included in meta-analysis, followed by a sensitivity analysis removing those studies most at risk of bias (classified as critical in ROBINS-I or high in RoB2).

With the available data and small number of studies, it was not possible to perform subgroup analysis for size of endometriomata, laterality and surgical approach as described in our original protocol.

Egger’s test was performed for all meta-analyses to assess for publication bias. Corresponding funnel plots were to be included in appendix if significant bias was identified, however no significant publication bias was seen for any of the meta-analyses performed.

## Results

### Study selection

**Fig. 1:**
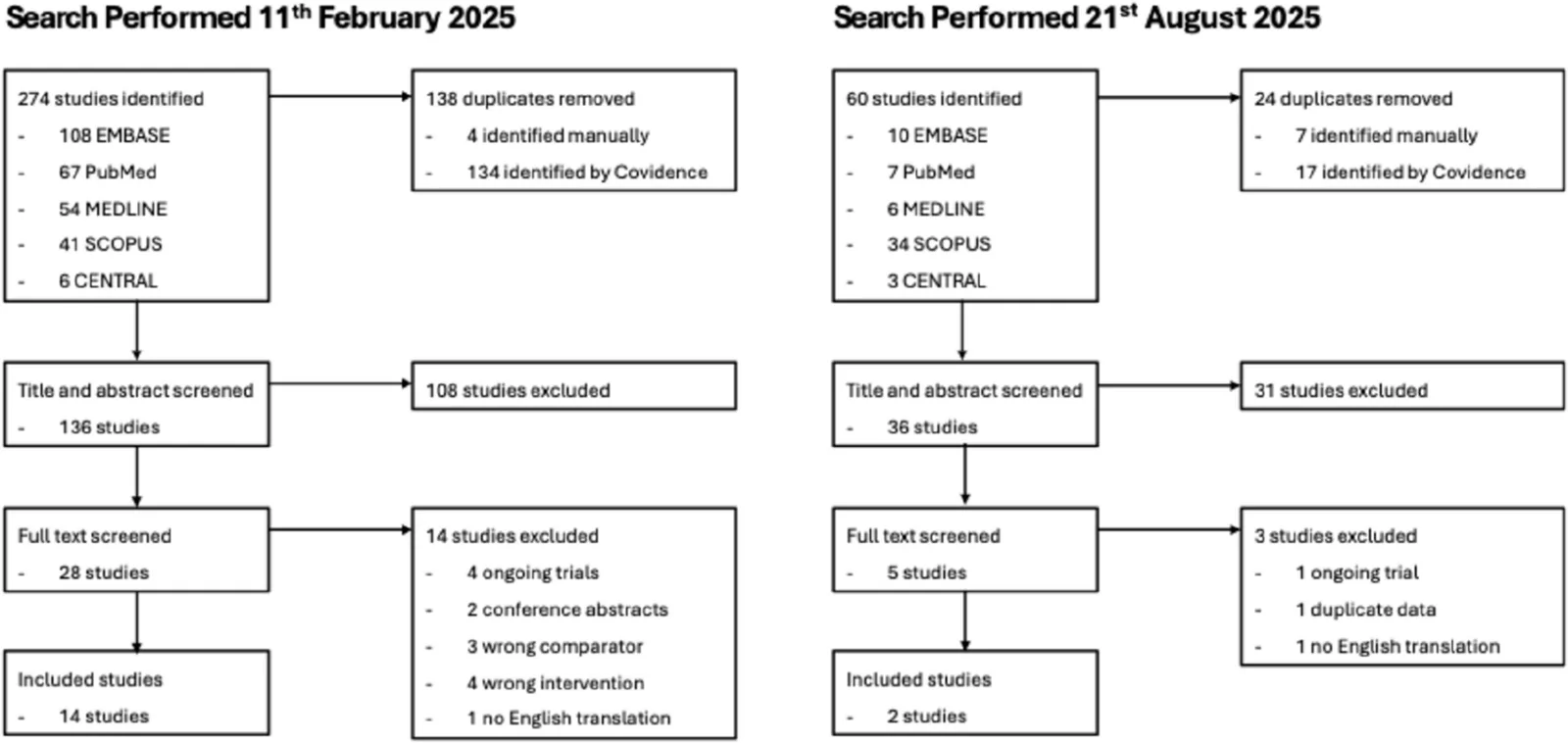
PRISMA flowcharts for initial search (left) and second search (right)

At the first search, 136 articles were identified using the described search criteria. Of these, 108 were excluded on review of title and abstract leaving 28 articles for full text review. Of these, 14 studies were excluded (see Fig. 1 for PRISMA flow chart) leaving 14 studies to be included. Where full text could not be obtained, or for ongoing trials, the authors were contacted. Only one author replied [50], who provided a full article awaiting publication for data previously reported at a conference. This was included in the systematic review and meta-analysis and has since been published [51]. One abstract appeared to fit criteria, however the full text was only available in Japanese [52], which we were unable to translate, so it was excluded. Two studies [53, 54] compared cystectomy to ‘aspiration with or without alcohol fixation’, both authors were contacted to request separate data for aspiration and sclerotherapy, neither responded.

A second search was performed prior to submitting for publication using the same search criteria but limiting to publications after 11th February 2025. This identified 36 further papers to screen, two of which were suitable for inclusion, one of which has been released as a pre-print awaiting publication [55]. Again, one abstract appeared to fit criteria however no English translation was available for the full text.

### Study characteristics and risk of bias

**Fig. 2:**
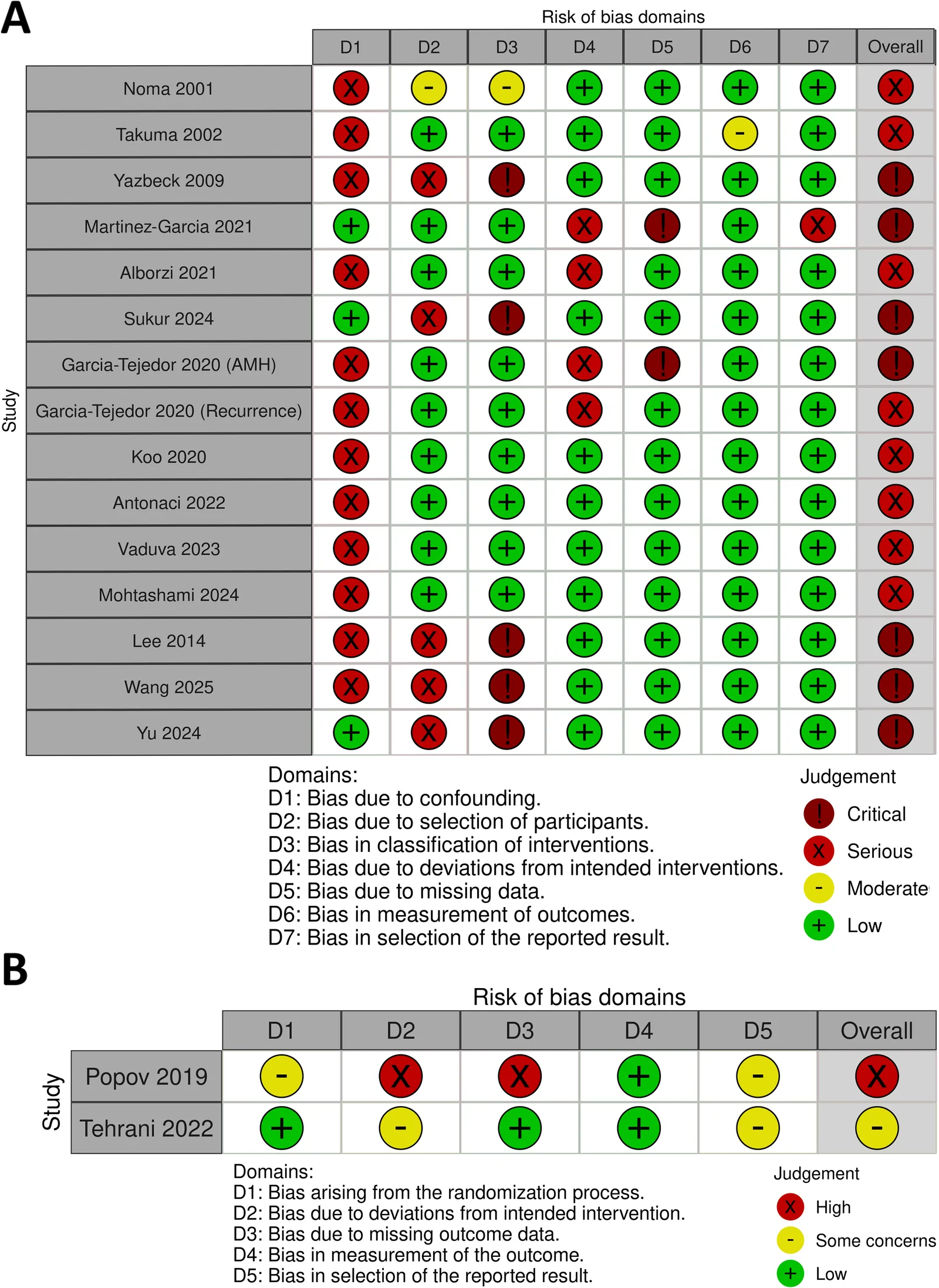
**A** ROBINS-I (where risk of bias varies depending on outcome the risk of bias has been given for each outcome) [67]. Figure **B** ROB-2 (where risk of bias varies depending on outcome the risk of bias has been given for each outcome) [67]

Characteristics of selected studies are summarised in Table 1 and outcomes of risk of bias analysis for these studies are summarised in Fig. 2A and B.

**Table 1:** Table of study characteristics

Study ID	Date of publication	Country	Study Design	Funding	Participant age range (years)	Cyst diameter	Cyst laterality	Treatment of concomitant endometriosis lesions	Sclerotherapy (n)	Ethanol concentration and instillation time	Control	Primary outcome (AMH)	Secondary Outcome
Noma 2001 [56]	2001	Japan	Retrospective cohort study	Not reported	No limit stated	> 2 cm	Not reported	Not clearly documented	TVUS guided (74)	100%, Flushed 1–2 times	Laparoscopic cystectomy (*n* = 26)	N/A	Recurrence, complications, CPR
Takuma 2002 [57]	2002	Japan	Retrospective cohort study	Not reported	No limit stated	Not reported	Not reported	In both groups: Adhesiolysis and electrocoagulation of peritoneal disease	Laparoscopic (25)	100%, 15 min	Laparoscopic cystectomy (*n* = 98)	N/A	Number of oocytes, CPR
Yazbeck 2009 [58]	2010	France	Prospective cohort study	Not reported	No limit stated	2–6 cm	Both unilateral and bilateral	Not clearly documented	TVUS guided (31)	100%, Max. 10 min	Laparoscopic cystectomy (*n* = 26)	N/A	Recurrence, complications, Number of oocytes, mature oocytes, CPR
Lee 2014 [59]	2014	Korea	Retrospective cohort study	Not reported	20–45	> 3 cm	Not reported	Not clearly documented	TVUS guided (29)	20%, Flushed 2–3 times	Laparoscopic cystectomy (*n* = 36)	N/A	Number of oocytes, mature oocytes, CPR, LBR
Garcia-Tejedor 2020 [60]	2020	Spain	Prospective cohort study	Instituto de Salud Carlos III	18–40	3.5–10 symptomatic, 5–10 cm asymptomatic	Both unilateral and bilateral for cystectomy. Only unilateral for sclerotherapy	Cystectomy only: adhesiolysis to mobilise ovary as required	TVUS or TAUS Guided (17)	100%, 15 min	Laparoscopic cystectomy (*n* = 14)	No significant difference	Recurrence, complications
Martinez-Garcia 2021 [61]	2021	Spain	Prospective cohort study	Instituto de Salud Carlos III, European Regional Development fund	18–40	3.5–10 symptomatic, 5–10 cm asymptomatic	Both unilateral and bilateral	Not clearly documented	TVUS or TAUS Guided (16)	100%, 15 min	Laparoscopic cystectomy (*n* = 10)	No significant difference	CPR
Alborzi 2021 [62]	2021	Iran	Prospective cohort study	Not reported	≤ 40	3–6 cm	Unilateral	In cystectomy group only: adhesiolysis and fulgaration of peritoneal disease	TVUS guided (44)	96%, left in situ	Laparosopic cystectomy (*n* = 57)	N/A	Recurrence, complications, Number of oocytes, mature oocytes, CPR, LBR, symptoms
Koo 2021 [63]	2021	Korea	Retrospective cohort study	A Grant from Yonsei University College of Medicine	No limit stated	3–10 cm	Both unilateral and bilateral	Not clearly documented	TVUS or TAUS guided (20)	99%, 20 min	Laparoscopic cystectomy (*n* = 51)	significant reduction in cystectomy group	Recurrence, complications
Antonaci 2022 [64]	2022	Italy	Retrospective cohort study	Not reported	21–38	3–10 cm	Both unilateral and bilateral	Cystectomy group only: adhesiolysis at ovarian fossa only	TVUS guided (23)	98%, 10 min	Laparosopic cystectomy (*n* = 26)	significant reduction in cystectomy group	Recurrence, complications, Number of oocytes, mature oocytes, CPR, LBR, symptoms
Vaduva 2023 [37]	2023	Romania	Retrospective cohort study	No external funding	20–35	3–8 cm	Unilateral	Not clearly documented	TVUS guided (54)	96%, 7 min	Laparoscopic cystectomy (*n* = 42)	significant reduction in cystectomy group	Recurrence, symptoms
Mohtashami 2024 [47]	2024	Iran	Retrospective cohort study	No external funding	25–38	≥ 3 cm	Both unilateral and bilateral	Cysterctomy only: thorough disection of ovarian adhesions, no documentation regarding pelvic endometriosis lesiosn	TVUS guided (44)	98%, 15 min	Laparoscopic cystectomy (*n* = 44)	significant reduction in cystectomy group	Recurrence, complications, symptoms
Sukur 2024 [50]	(2024) Awaiting peer review	Turkey	Retrospective cohort study	Not reported	20–40	≥ 4 cm	Both unilateral and bilateral	Not clearly documented	TVUS guided (20)	96.5%, 10 min	Laparoscopic cystectomy (*n* = 31)	significant reduction in cystectomy group	Recurrence, complications, Number of oocytes, mature oocytes, CPR, LBR
Popov 2019 [65]	2019	Russia	Randomised controlled trial	Not reported	25–35	1.5–10 cm	Unilateral	Both groups: adhesiolysis, excision and coagulation of pelvic endometriosis	laparoscopic (18)	70%, 2 min	Laparoscopic cystectomy (*n* = 18)	significant reduction in cystectomy group	Recurrence, CPR symptoms
Tehrani 2022 [36]	2022	Iran	Randomised controlled trial	Grants from Isfahan University of Medical Sciences	18–40	4–10 cm	Both unilateral and bilateral	Cystectomy only: excision of abdominal endometrial lesions	TVUS guided (35)	95%, 20 min	Laparoscopic cystectomy (*n* = 35)	significant reduction in cystectomy group	Recurrence
Yu 2025 [66]	2025	China	Retrospective cohort	Joint funds of the National Science Foundation of China and the Pioneer and Leading Goose R&D Orogramn Zhejiang	≤ 40	No limit stated	Both unilateral and bilateral	Excision, stripping or coagulation techniques used to manage other pelvic endometriotic lesions	Laparoscopic (33)	100%, 5–7 min	Laparoscopic cystectomy (34)	significant reduction in cystectomy group	Number of oocytes, CPR
Wang 2025 [55]	(2025) Awaiting peer review	China	Retrospective cohort	Joint funds by the Natural Science Foundation of Guangdong Province,Medical Science and Technology Research Foundation of Guangdong Province, Guangzhou Science and Technology Plan Project, and Shenzhen Baoan District Medical and Health Research Project	≤ 41	No limit stated	Not reported	Adhesiolysis and restore anatomy fo both laparoscopic arms. Visible pelvis endometriosis lesions were electrocoagulated	Laparoscopic (47) TVUS Guided (91)	100%, 5–7 min	Laparoscopic cystectomy (69)	Significant reduction in cystectomy group	Recurrence, complications, Number of oocytes, mature oocytes, CPR

*TVUS* transvaginal ultrasound, *TAUS* transabdominal ultrasound, *CPR* Clinical pregnancy rate, *LBR* Live birth rate

### Risk of Bias

Risk of bias assessments: Fig 2.

### Anti-Mullarian Hormone (AMH)

Ten studies described AMH levels before and after intervention, 8 of these were cohort studies and 2 were randomised controlled trials. The 10 studies include 757 patients. In a sensitivity analysis removing studies at high/critical risk of bias, 5 studies with 374 patients remained (Table 2).

**Table 2:** Table of AMH results

Study	Sclerotherapy method	AMH level sclerotherapy (ng/mL)					Sclerotherapy (n)	AMH level cystectomy (ng/mL)					Cystectomy (n)	*P* value (Sclerotherapy compared to Cystectomy)
		Baseline	1 month	3 months	6 months	*P* value		Baseline	1 month	3 months	6 months	*P* value		
Wang 2025 [55]	Laparoscopic 100% 5–7 min	3.63 ± 1.92	3.52 ± 1.81				47	3.69 ± 1.73	2.35 ± 1.87				69	Baseline: 0.932 1 month: 0.040
	TVUS guided 100% 5–7 min	3.79 ± 2.06	3.09 ± 1.84				91							
	Combined sclerotherapy	3.74 ± 2.01	3.23 ± 1.83				138							
Koo 2021 [63]	USS guided 99% 20 min	*2.3* *[1.1,5.3]*			*2.6* *[0.9,4.9]*	0.243	20	3.00 [1.3,5.5]			1.6 [0.7,3.2]	< 0.001	51	Baseline: 0.766 6 months: ND
Antonaci 2022 [64]	TVUS guided 98% 10 min	3.21 ± 2.43		3.24 ± 2.45	3.17 ± 2.15		23	3.14 ± 2.45		2.18 ± 2.10	2.22 ± 1.97		26	baseline: 0.22 3 months: 0.03 6 months: 0.045
Vaduva 2023 (20–29) [37]	TVUS guided 96% 7 min	3.24 [1.89,4.59]		3.05 [1.51,4.58]		0.1873	12	3.18 [1.83, 5.3]		1.62 [0.74, 3.08]		< 0.001	8	
Vaduva 2023 (30–35) [37]	TVUS guided 96% 7 min	2.26 [1.1–3.42]		2.18 [1.08,3.28]		0.2029	42	2.38 [1.14,3.42]		1.16 [0.26,2.17]		< 0.001	34	
Sukur 2024 [50]	TVUS guided 96.5% 10 min	2.6 ± 1.5			2.3 ± 1.5	0.576	20	1.9 ± 0.9			0.9 ± 0.6	0.001	31	
Mohtashami 2024 [47]	TVUS guided 98% 15 min	1.14 ± 1.53			1.00 ± 0.99		44	1.46 ± 1.07			0.87 ± 1.23		44	Baseline: 0.137 6 months: 0.092
Garcia-Tejedor 2020 [60]	USS guided 100% 15 min	2.20 [1.23,3.74]			2.02 [0.90,3.14]		Baseline: 16 6 months: 12	1.09 [0.82,3.33]			1.35 [0.60,1.61]		Baseline: 10 6 months: 9	Baseline: NS 6 months: NS
Popov 2019 [65]	Laparoscopic 70%, 2 min	3.4 ± 0.7		2.9 ± 0.4		NS	Baseline: 18 3 months: 15	3.1 ± 0.5		1.8 ± 0.24		≤ 0.05	Baseline: 18 3 months: 16	
Tehrani 2022 [36]	TVUS guided 95% 20 min	2.12 ± 1.05		2.09 ± 1.01		< 0.120	35	2.48 ± 1.34		1.62 ± 1.22		< 0.001	35	
Yu 2025 [66]	Laparoscopic 100% 5–7 min	1.32 (0.84,1.86)		1.13 (0.59,1.86)		0.098	33	1.52 (1.02,1.81)		1.15 (0.76,1.67)		0.006	34	baseline: 0.60 3 months: 0.218

AMH results reported by study: In cases where median and interquartile range were provided in the original data, these were converted to an estimate of mean and standard deviation using the formula provided by Wan et al. (48). ND = not documented, NS = non-significant. SD ± CI, Median [IQR]. AMH assay not given for any study

**Fig. 3:**
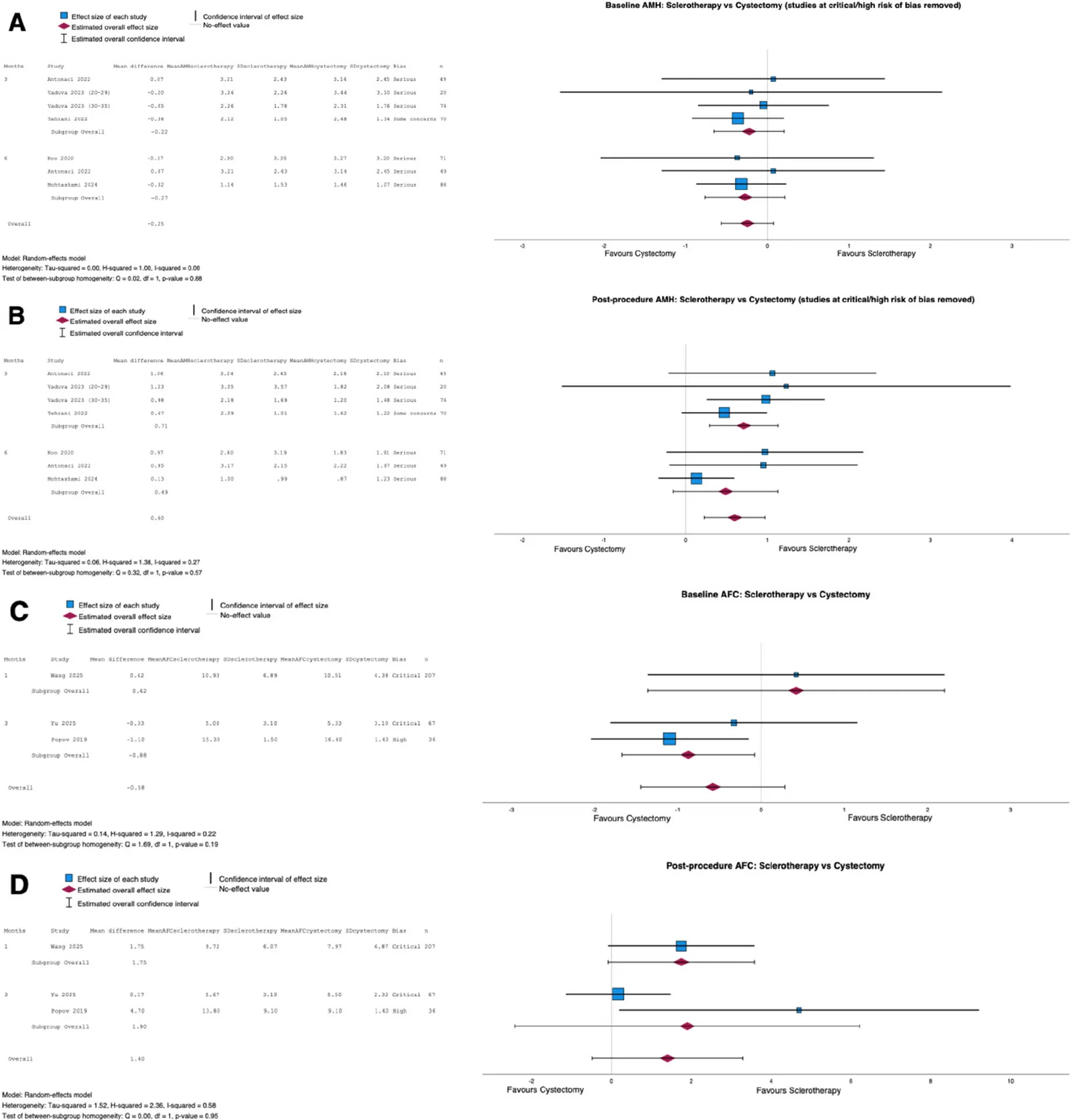
**A**-**D**: Forest plots relating to measures of ovarian reserve. Figure 3A: Baseline AMH (removing high/critical bias) MD −0.25, CI −0.57 to 0.08. Figure 3B: Post procedure AMH (removing high/critical bias) MD 0.60, CI 0.22 to 0.97, *p* = 0.002. Figure 3C: AFC at baseline MD –0.58, CI –1.45 to 0.29,* p* = 0.189. Figure 3D: AFC post procedure MD 1.40, CI –0.49 to 3.30, *p* = 0.146

When all studies were included in the meta-analysis there was no significant difference in AMH at baseline between the sclerotherapy and cystectomy arms (mean difference (MD) 0.02, CI –0.20 to 0.25, *p* = 0.830, see Additional File 2). This remained non-significant in the sensitivity analysis, with a trend toward higher AMH levels in the cystectomy arm (MD −0.25, CI −0.57 to 0.08, *p* = 0.136, see Fig. 3A).

Post procedure, AMH was significantly higher in the sclerotherapy arm when all studies were included in the analysis (MD 0.75, CI 0.43 to 1.06, *p* < 0.001, see Additional File 3) as well as when studies at critical/high risk of bias were removed (MD 0.60, CI 0.22 to 0.97, *p* = 0.002, see Fig. 3B). Subgroup analysis did not show any significant trend relating to AMH over time post procedure.

When comparison was made between AMH pre- and post-procedure for each study arm, a significant reduction in AMH was seen following sclerotherapy (MD –0.27, CI –0.45 to –0.08, *p* = 0.006) which became non-significant when studies assessed as at high/critical risk of bias were removed (MD −0.07, CI −0.38 to 0.23, *p* = 0.63). Following cystectomy, a significant reduction in AMH was seen post procedure (MD −0.93, CI −1.21 to −0.65, *p* < 0.001which remained significant when studies assessed as high/critical risk of bias were removed (MD −0.86, CI −1.16 to −0.56, *p* < 0.001). Again, subgroup analysis did not show any significant trend relating to AMH over time post procedure (See Additional Files 4–7 for forest plots).

### Antral follicle count (AFC)

Six studies reported on AFC, with only four measuring AFC both before and after the intervention. One study [61] provided a graphical representation of the change which the authors describe as non-significant but no numerical data was provided. Two studies noted a reduction in AFC post procedure in the cystectomy arm but not in the sclerotherapy arm [55, 65]. While one study found no change in AFC post procedure in either treatment arm [66]. Two studies described AFC at the time of IVF with both describing a significantly lower AFC in patients with a history of cystectomy compared to those with a history of sclerotherapy, these included Lee [59] (8.2 ± 3.9 vs 11.1 ± 3.8, *p* = 0.024) and Sukur [50] (5.4 ± 2.9 vs 10.7 ± 5.4, *p* < 0.001).

Pooling of data showed no significant difference in AFC between each intervention either pre- or post-procedure (MD –0.58, CI –1.45 to 0.29, *p* = 0.189 and MD 1.40, CI –0.49 to 3.30, *p* = 0.146 respectively, see Fig. 3 C-D). Importantly, all 6 studies were considered to be at high or critical risk of bias making it difficult to draw conclusions from these findings.

### Recurrence

**Fig. 4:**
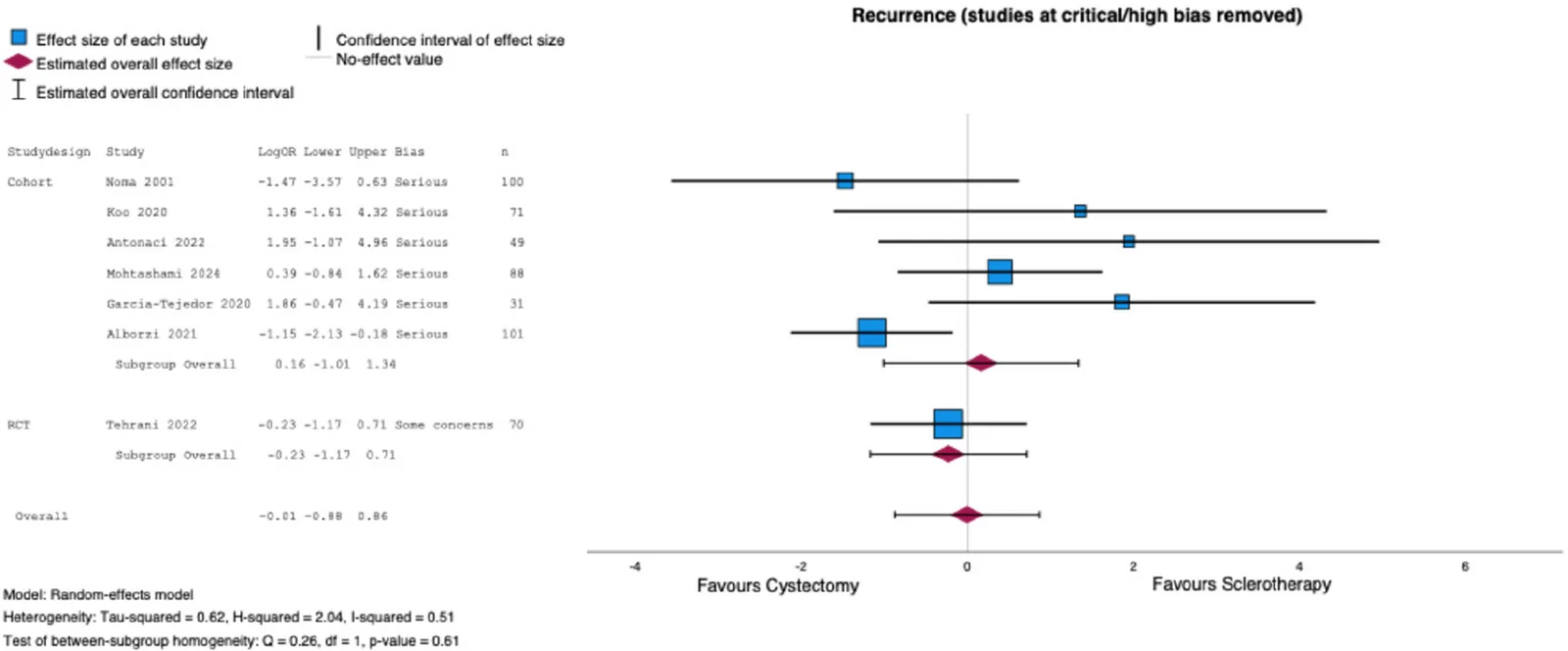
Recurrence (Critical/High Bias removed): LogOR −0.01, CI –0.88 to 0.86, *p* = 0.990

Ten studies (804 participants) reported on recurrence in both treatment arms (See Additional File 8 for table of values). In two studies no recurrence was identified in either arm. Overall, no significant difference in recurrence was seen (LogOR 0.28, CI –0.57 to 1.12, *p* = 0.523, See Additional File 9). In a sensitivity analysis, removing studies at critical/high risk of bias, the result remained non-significant (LogOR −0.01 CI −0.88–0.86, *p* = 0.99, see Fig. 4). All remaining studies were assessed as at serious risk of bias or with some concerns.

### Complications

Complications were reported in 9 studies, however as the method of reporting and follow up varied between studies they have been summarised in a tabular fashion (See Table 3). Overall, only one serious complication was reported following sclerotherapy out of 411 patients (0.24%). Two serious complications were seen in 261 patients undergoing cystectomy (0.76%).

**Table 3:** Table of complications: serious = Clavien Dindo III-IV or described as serious by authors, N/A = not applicable. LST = laparoscopic sclerotherapy, UST = US guided sclerotherapy

Study ID	Sclerotherapy			Cystectomy			Follow up period
	Total Complications n (%)	Serious complications (n)	Details	Total Complications n (%)	Serious complications (n)	Details	
Noma 2001 [56]	20 (27%)	0	4 abdominal pain, 3 alcohol intoxication, 13 mild abnormal bleeding	Not reported	N/A	N/A	Unclear
Yazbeck 2009 [58]	1 (3.2%)	0	Acute abdominal pain	Not reported	N/A	N/A	Unclear
Garcia-Tejedor 2020 [60]	2 (11.7%)	0	Not documented	4 (28.6%)	1	Intestinal occlusion requiring repeat surgery	6 weeks
Koo 2021 [63]	0 (0%)	0	N/A	2 (3.9%)	0	2 wound infections	6 months
Alborzi 2021 [62]	Not reported	0	N/A	Not reported	0	N/A	Unclear
Antonaci 2022 [64]	2 (8.7%)	0	Post operative pain, vaginal infection	6 (23.1%)	1	3 post operative pain, 1 surgical site infection, 1 ovarian abscess requiring return to theatre	Mean 37.5 months
Sukur 2024 [50]	2 (10%)	0	1 pain, 1 fever requiring intravenous antibiotics	Not reported	N/A	N/A	Within 3 months for sclerotherapy
Mohtashami 2024 [47]	1 (2.3%)	1	Ovarian abscess	0 (0%)	0	N/A	Unclear
Wang 2025 (LST) [55]	0 (0%)	0	N/A	0 (0%)	0	N/A	1 month
Wang 2025 (UST) [55]	0 (0%)	0	N/A				

### Symptoms

The impact on symptoms in both intervention groups was reported in 5 studies, however there was a wide variety in methods of reporting and in the symptoms described.

Garcia-Tejedor et al. [60] measured pain using the visual analogue scale (VAS) however reported the results descriptively. They describe no significant difference in pain improvement between arms but provide no quantitative data. Koo et al. [63] also used the VAS; they report a significant decrease in pain scores in the sclerotherapy group (5.79 ± 1.36 to 1.26 ± 0.87, *p* = < 0.001) and no significant difference in rates of symptom relief (*p* > 0.999) between sclerotherapy and cystectomy. The details of any decrease in pain score were not given for the cystectomy arm. Mohtashmi et al. [47] reported VAS at baseline and at 2, 6 and 12 months follow up. In both interventions a significant decrease in pain score was seen at all time points compared to baseline for both dysmenorrhoea and dyspareunia. No comparison was made between the two interventions. Pain was reported by Popov et al. [65], with a VAS out of 10 performed before surgery and after restoration of menstruation. An improvement in pain scores was seen in both sclerotherapy and cystectomy however p values were not reported.

Antonaci et al. [64] reported on an impression of global improvement (PGI-I) at 3 months post treatment. In both groups the majority reported the treatment to be a success with no significant difference seen between each intervention.

Overall, both treatment arms appear to provide symptomatic improvement with neither showing a benefit over the other.

### Pregnancy and reproductive outcomes

#### Oocytes retrieved

**Fig. 5:**
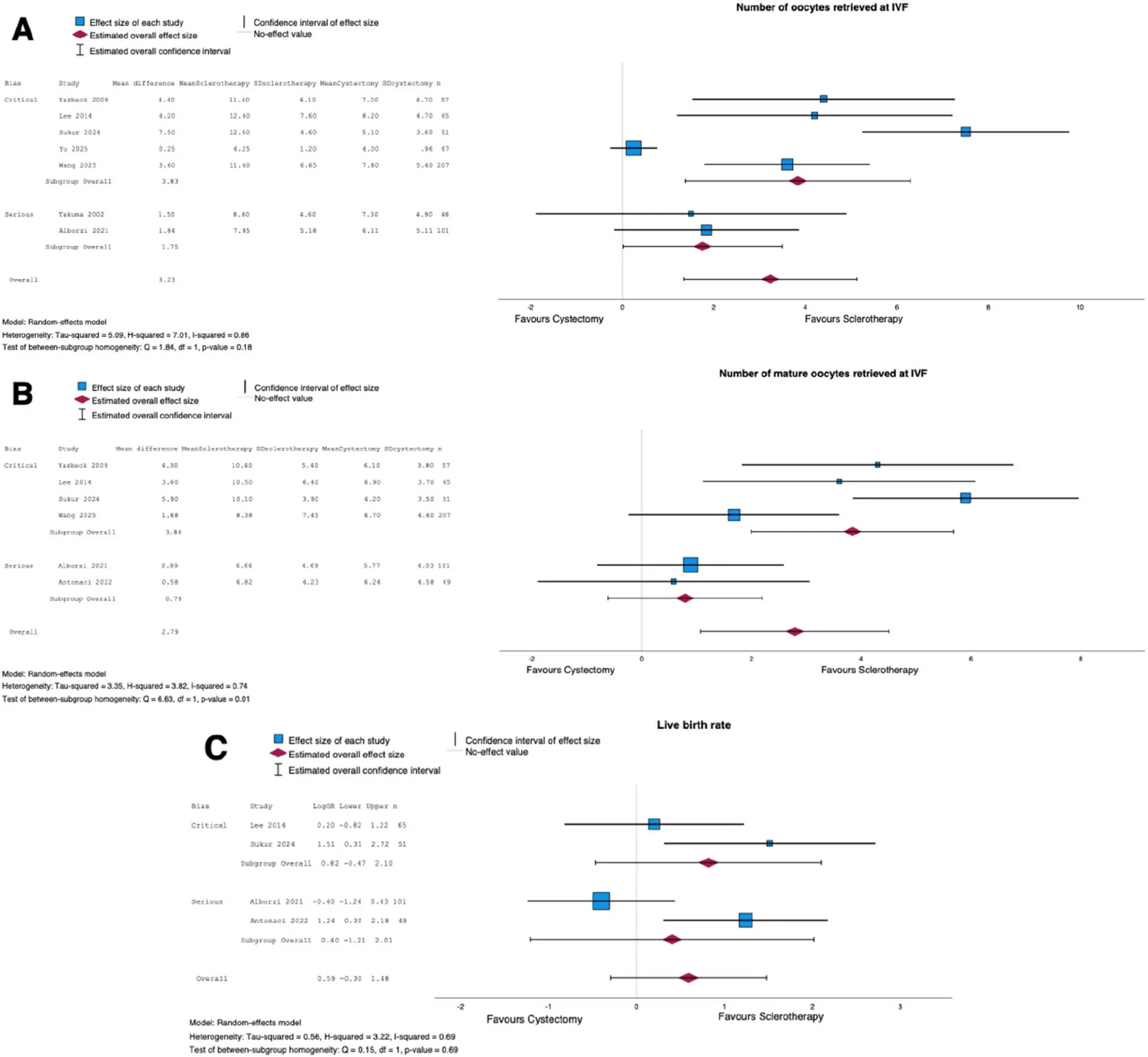
**A**-**C** Forest plots relating to pregnancy outcomes. Figure 5**A**: Forest plot of Number of oocytes retrieved. Figure 5**B**: Forest plot of Number of mature oocytes (MII) retrieved. Figure 5**C**: Forest plot for live birth rate

Six studies (594 participants) reported on mean number of oocytes retrieved (see Additional File 10 for table of values). Meta-analysis showed a significantly higher number of oocytes retrieved in the sclerotherapy arm (MD 3.23, CI 1.34 to 5.12, *p* < 0.001) and this remained significant in the sensitivity analysis when studies at critical risk of bias were removed (MD 1.75, CI 0.01 to 3.49, *p* = 0.048). However, the confidence interval approaches zero (See Fig. 5A).

#### Mature oocytes

Six studies (530 participants) reported on mature (metaphase II) oocytes, (see Additional File 10 for table of values). Meta-analysis of these studies initially showed a significantly higher number of mature oocytes retrieved in the sclerotherapy group (MD 2.79, CI 1.08 to 4.51, *p* = 0.001). However, in the sensitivity analysis when studies with critical level of bias were removed the result becomes non-significant (MD 0.79, CI −0.61–2.19, *p* = 0.27) (See Fig. 5B).

#### Pregnancy rate

Eleven studies reported on pregnancy rate, however the definition of clinical pregnancy rate varied widely and in some cases was not reported. Meta-analysis was therefore not appropriate and the findings from each study are reported in Table 4.

**Table 4:** Table of clinical pregnancy rate. If p value reported as non-significant it was because true value not provided in paper. FH = fetal heart, GS = gestational sac, USS = ultrasound scan

Study ID	Method of conception	Definition of clinical pregnancy rate	Pregnancy events		*p* value
			Sclerotherapy n (%)	Cystectomy n (%)	
Noma 2001 [56]	Not specified	Not defined	12 (52.2%)	8 (61.5%)	Non-significant
Popov 2019 [65]	Spontaneous conception	Not defined	8 (44.4%)	6 (33.3%)	Not reported
Martinez-Garcia 2021 [61]	Spontaneous conception	Not defined	3 (42.9%)	0 (0%)	Not reported
Takuma 2002 [57]	Spontaneous	Not defined	5 (33.3%)	16 (25.8%)	Not reported
Takuma 2002 [57]	IVF	Cumulative, not otherwise specified	10 (70%)	18 (50%)	Not reported
Yazbeck 2009 [58]	IVF	FH seen at 12 weeks, pregnancy rate per cycle	14 (48.3%)	5 (19.2%)	0.04
Lee 2014 [59]	IVF	GS seen on USS, pregnancy rate per cycle	12 (41.4%)	13 (36.1%)	Non-significant
Alborzi 2021 [62]	IVF	GS seen at 6 weeks, pregnancy rate per patient	15 (34.1%)	24 (42.1%)	0.41
Antonaci 2022 [64]	IVF	FH seen at 7 weeks, pregnancy rate per cycle	25 (48.1%)	11 (19.6%)	0.03
Sukur 2024 [50]	IVF	FH seen at 7 weeks, cumulative pregnancy rate per cycle	13 (65%)	10 (32.3%)	0.035
Yu 2025 [66]	IVF	GS seen on TVUS at 6–8 weeks per transplant cycle	20 (60.6%)	12 (36.4%)	< 0.05
Wang 2025 (LST) [55]	IVF	FH seen at 30 days post embryo transfer per cycle	26 (78.8%)	36. (62.1%)	0.100
Wang 2025 (UST) [55]	IVF	FH seen at 30 days post embryo transfer per cycle	55 (72.4%)		0.206

#### Live birth rate

Four studies reported on live birth rate, all of which were following IVF and embryo transfer (see Additional File 10 for table of values). In 2 of these studies [50, 64] this was clearly defined as live birth over 22 weeks gestation, in the remaining studies no definition was provided. No significant difference was seen in live birth rate between each treatment arm (LogOR 0.59, CI −0.30 to 1.48, *p* = 0.192), The results remained non-significant when studies of critical/high risk of bias were removed (LogOR 0.40, CI –1.21 to 2.02, *p* = 0.62) (See Fig. 5C).

## Discussion

### Findings

#### AMH

The detrimental effect of cystectomy on AMH in the treatment of endometriomata has been well documented [23, 68]. While ethanol sclerotherapy is generally considered to have a less negative effect on ovarian reserve, the magnitude of this effect is unclear. Some studies have found no difference in AMH before and after sclerotherapy treatment [35, 38], whereas others have noted a significant reduction in AMH [69], although the decline is less than that reported for cystectomy. Importantly, these were single arm studies without a direct comparator. Our findings, that post procedure AMH levels are higher following sclerotherapy compared to cystectomy, and that sclerotherapy does not cause a significant reduction in AMH when used in the management of endometriomata, are consistent with those of a previous meta-analysis [44], which pooled the data from 4 studies, including single arm studies. Importantly our meta-analysis only includes studies with a comparator, and shows a benefit to sclerotherapy in direct comparison to the standard treatment of laparoscopic cystectomy with regards to ovarian reserve.

However, there are some reasons for caution. We had initially planned to compare changes in ovarian reserve from baseline but only one study [47] reported this, with all others reporting on mean pre- and post-procedure measures, which does not account for individual variation in AMH or regression to the mean. Since change from baseline could not be calculated from the reported data, our analysis also had to rely on comparing mean pre- and post-procedure measures, a major limitation. We would encourage investigators to report the more meaningful change from baseline in future studies to control for inter-individual variations in AMH and to support synthesis of data between studies.

While AMH is a useful marker of ovarian ageing and can be used to predict the ovarian response to stimulation [70], it is important to remember it is a measure of oocyte quantity, not quality, and as such cannot predict the likelihood of an ongoing pregnancy. While AMH levels are a useful addition to discussions regarding fertility and family planning, more research is required to understand how endometriosis contributes to subfertility.

#### AFC

Both AFC and AMH have been shown to be lower in patients with endometriosis, particularly those with endometriomata, compared to controls [71]. However, the use of AFC as a measure of ovarian reserve in the presence of endometriomata has been questioned [22]. In the absence of endometriomata, AFC and AMH are strongly positively correlated [72]. However, there is evidence that following cystectomy for endometriomata when AMH levels drop, AFC remains stable [23, 68, 73]. A retrospective study comparing AFC with number of oocytes retrieved, in women with unilateral endometriomata undergoing ART, found that despite the AFC being lower in the ipsilateral ovary, the number of retrieved oocytes was similar [74]. As a result, AFC may not be a reliable marker of ovarian reserve in the presence of endometriomata. In our analysis, although six studies described AFC, only four of those studies report AFC before and after intervention, the results were conflicting, and all studies were assessed as at a critical/high level of bias making it difficult to draw any reliable conclusions.

#### Recurrence

One of the concerns with adopting ethanol sclerotherapy has been recurrence rates, which have been reported as high as 62.5% [31] in cases where ethanol was instilled for < 10min. Recurrence rates were much lower, ranging from 0–13.3% when ethanol was left in situ [31]. This was supported by a meta-analysis examining the impact of ethanol instillation time on recurrence, which found recurrence rates were significantly higher if ethanol was instilled for ≤ 10 min [44]. Our findings, that there is no significant difference in recurrence rates between ethanol sclerotherapy and cystectomy, were consistent with those previously reported [43]. It is important to note that recurrence was reported in a variety of ways by different studies, and the follow up period ranged from a few months to 7 years. Only one study showed significantly increased rates of recurrence in the sclerotherapy arm of their prospective study [62]. This is potentially surprising considering they left ethanol in situ. The authors attributed this to their longer follow up period [62], however it is important to consider the disparity between each intervention. The treatment arm underwent ultrasound guided sclerotherapy, while the comparator underwent laparoscopic cystectomy and fulguration of active endometriosis. Given that one arm received treatment for all pelvic endometriosis, while the other only received treatment of the endometriomata with pelvic disease untouched, it is hardly surprising the recurrence rates were higher in the latter, especially over the long follow up period when active disease could allow for new cysts to develop.

Although our analysis found no significant difference in recurrence rates, it is important to consider the wide variety in study design with different instillation times, definitions of recurrence and follow up periods between studies. Further well-designed studies, ideally randomised controlled trials using standardised definitions, are required to assess this further.

#### Complications

When counselling women on management, it is important to provide detailed information on the risks as well as the benefits for each treatment option. Unfortunately, despite 9 studies reporting on complications, only 5 studies reported complications in both study arms. There was also a lack of clarity regarding follow up times and the definition of a serious complication. Only two studies used a standardised grading system for classifying complications [60, 64], both utilising Clavien-Dindo classification, which helps to standardise findings and should be considered in future studies. It is important to note that most studies reporting on complication rates used ultrasound guided sclerotherapy and may not be directly applicable to laparoscopic sclerotherapy, especially in cases with DIE and anatomical distortion that might complicate surgical access. With these caveats in mind, it is difficult to make a definitive statement about the relative safety of ethanol sclerotherapy and laparoscopy, but in our analysis the serious complication rates for both were low, in common with previous studies [31, 69].

#### Symptoms

Endometriosis is known to have a significant impact on quality of life [4, 8] with pain being one of the main indications for treating endometriomata. As such, it is vital that any offered treatment provides appropriate symptom control. The method of reporting on pain symptoms varied widely between studies, and while the majority used VAS to score the pain [47, 60, 63, 65], the results were not presented in a way that allowed data synthesis. Importantly, all the studies reporting on symptoms described a symptomatic improvement with ethanol sclerotherapy, with no difference seen when comparison was made to cystectomy. While VAS is a validated scale often used both clinically and in research to measure pain [75], it does not provide any insight into how this pain, or other symptoms, might impact quality of life. In view of this, the Endometriosis Health Profile (EHP-30) has been developed to measure health related quality of life [76] in endometriosis and may prove useful for enabling pooled analysis if widely adopted going forward.

It is important to consider that most studies included in this systematic review and meta-analysis compare ultrasound guided sclerotherapy to laparoscopic cystectomy, and there was wide variation and lack of clarity as to what treatment of any concomitant endometriosis was performed in the cystectomy arm. In addition, few of the studies report on whether hormonal treatment was used post operatively, which could impact the findings with regards to symptoms. While the results regarding symptom improvement are encouraging, further studies using validated scales for symptom reporting and taking account of the effects of any concomitant surgical or pharmacological intervention are needed.

#### Pregnancy outcomes

While our analysis suggests sclerotherapy may not cause the reduction in AMH seen following cystectomy, it is important to consider the practical implications of this, with the delivery of a healthy baby the main goal for patients with fertility wishes. In their meta-analysis, Cohen et al. found significantly more oocytes were retrieved following sclerotherapy compared to cystectomy, but pregnancy rates were similar across both groups [31]. Our analysis also found significantly more oocytes retrieved post sclerotherapy (MD 1.75, CI 0.01 to 3.49, *p* = 0.048) although the confidence intervals approached zero. However, there was no significant difference in the number of mature oocytes retrieved between treatment arms (MD 0.79, CI −0.61–2.19, *p* = 0.27). This fits with our findings that AMH is better preserved following sclerotherapy so maintaining oocyte quantity. However, the presence of endometriosis, and endometriomata, may have a negative impact on the oocyte quality as demonstrated by our findings regarding the number of mature oocytes. Importantly, for both oocyte and mature oocyte numbers, only two studies remained for analysis after the removal of studies most at risk of bias. Further well designed, adequately powered studies are required to either confirm or refute these findings.

Unfortunately, due to the varied method of defining and reporting clinical pregnancy rates in the studies analysed, it was not appropriate to pool the results comparing sclerotherapy to cystectomy. Excluding those studies at high or critical levels of bias, two studies [56, 62] found no difference in pregnancy rates while two studies found clinical pregnancy rate to be significantly higher in the sclerotherapy group [57, 64]. Interestingly, Alborzi et al. [62] treated any peritoneal endometriosis at the time of cystectomy, whereas the sclerotherapy group did not receive any treatment for their pelvic endometriosis. Takuma et al. [57] were one of the few groups who performed laparoscopic sclerotherapy and treated peritoneal disease in both groups. Noma et al. [56] did not report on whether pelvic endometriosis was treated, and Antonaci et al. [64] did not treat pelvic endometriosis. The discrepancy in findings could be explained if both performing sclerotherapy and treating pelvic endometriosis contributed to improved pregnancy rates, it is important to note, however, that all studies were at best at serious risk of bias making it difficult to draw any reliable conclusions.

None of the previous meta-analyses included live birth rate in their analysis [31, 43–45]. It should be noted that all studies reporting on live birth rate in our analysis looked at conception following IVF and may not be applicable to spontaneous conception rates. We found that there was no difference in live birth rate between treatment arms following IVF suggesting there are factors other than ovarian reserve, and therefore oocyte quantity, that contribute to the reduced fertility seen in endometriosis patients. There are multiple theories as to how endometriosis may contribute to subfertility, such as the pro-inflammatory environment exerting its effect on the oocyte, sperm, fallopian tubes or endometrium, altered endometrial receptivity, or anatomical distortion [77–79]. More research is needed to fully understand these mechanisms, how they may contribute and if any intervention has a beneficial effect on pregnancy rates.

### Limitations

There were a number of general limitations to this analysis, as well as specific ones with respect to the primary outcome of AMH measurement. As discussed previously, a formal meta-analysis of between group change in AMH was not possible due to insufficient reporting of change score variance data, as such an exploratory between group pre- and post- analysis was performed instead. While our findings suggest sclerotherapy may have a lesser impact on ovarian reserve further evidence is required to confirm this hypothesis. In addition, only a small number of studies met the inclusion criteria and the included studies were mostly small, retrospective, observational studies with only two randomised controlled trials. Importantly, none of the included studies were categorised as being at low risk of bias, with the majority at serious risk of bias. In particular, many of the studies looking at pregnancy outcome showed significant selection bias by only looking at a population undergoing IVF [50, 55, 58, 65, 66] with no consideration for those women who conceived spontaneously following each intervention. This limits the ability to draw reliable conclusions from our findings and suggests the need for further well-designed studies, ideally randomised controlled trials, to confirm these results. While Eggers test did not identify any significant publication bias, its ability to detect bias is limited when the number and size of studies is small, and as such the publication bias in this study may be underestimated.

A significant limitation to the analysis was the substantial clinical heterogeneity between studies. Although the sclerotherapy procedure is similar between laparoscopic and ultrasound guided approaches, the former requires a general anaesthetic allowing for the excision of pelvic endometriosis and adhesiolysis, both of which may have impacted the findings. If excisional surgery or adhesiolysis was required at the site of the ovaries, this could have an impact on ovarian reserve, while if excision of concomitant pelvic endometriosis is not performed the occurrence of new cysts may be mislabelled as recurrence. In addition, there is wide variation in the method of sclerotherapy between studies with different instillation times and ethanol concentrations, all of which may impact the effectiveness of any chemical ablation and have a corresponding impact on the results. While the majority of the studies included both unilateral and bilateral endometriomata they did not differentiate between the two in reporting the results. Considering AMH reductions of 30–39.5% have been reported post unilateral cystectomy, while reductions of 44–57% were seen post bilateral cystectomy [22, 23], it is possible that intervention on both ovaries may have a greater impact on ovarian reserve than intervention on one ovary alone. The timing between intervention on the ovaries and initiation of IVF is another area of clinical heterogeneity that may impact results and in many of the studies the time interval was significantly different between each treatment arm, as well as varying between studies. The available data and small number of studies meant subgroup analysis was not feasible to assess for this impact. Finally, the range of definitions for clinical pregnancy rate varied between a gestational sac on ultrasound to a fetal heart at 12 weeks, in those that provided a definition, making it impossible to pool or compare findings between studies.

The performance of future meta-analyses will be facilitated if studies use standardised methods of reporting, such as validated questionnaires, e.g. EHP30, standardised classification systems, e.g. Clavien-Dindo classification, and agreed definitions for outcomes such as clinical pregnancy rate and recurrence. A lack of clarity in study design and reporting was apparent throughout, with none of the papers reporting the details of the AMH assay utilised and many not even defining endometriomata recurrence. Standardising definitions and methods of reporting would facilitate future data synthesis.

## Conclusions

It is difficult to make conclusions from the available data, given the concerns regarding bias and study design. While our exploratory analysis suggests that ethanol sclerotherapy for the treatment of endometriomata may maintain ovarian reserve compared to cystectomy, further well-designed studies are required to confirm this hypothesis and to draw conclusions regarding the secondary outcomes of recurrence, complications, symptomatology and pregnancy outcomes.

This analysis highlights the need for good quality evidence on this subject to support patients and their clinicians in decisions regarding management of endometriomata, particularly when fertility preservation is required.

## Supplementary Information

Additional file 1.

Additional file 2.

Additional file 3.

Additional file 4.

Additional file 5.

Additional file 6.

Additional file 7.

Additional file 8.

Additional file 9.

Additional file 10.

Additional file 11.

Additional file 12.

## Data Availability

The datasets used and/or analysed during the current study are available from the corresponding author on reasonable request.

## Abbreviations

AFC: Antral follicle count
AMH: Anti-Mullerian hormone
ART: Assisted reproductive techniques
DIE: Deep infiltrating endometriosis
EHP 30: Endometriosis Health Profile questionnaire
IVF: In vitro fertilisation
LST: Laparoscopic sclerotherapy
MD: Mean difference
PRISMA: Preferred Reporting Items for Systematic reviews and Meta-Analyses
PROSPERO: International prospective register of systematic reviews
RCT: Randomised controlled trial
ROB2: Revised Cochrane risk-of-bias tool for randomised trials, version 2
ROBIN-I: Risk of bias in Non-Randomised Studies of interventions, version 2
TA: Transabdominal
TV: Transvaginal
US: Ultrasound
UST: Ultrasound guided sclerotherapy
VAS: Visual analogue scale
