# Supplementary material for: Ethanol sclerotherapy versus cystectomy in the management of endometriomata: a systematic review and meta-analysis
Source: BMC Womens Health. 2026 Jul 18;26:518. doi: 10.1186/s12905-026-04645-2 (PMC13663109; doi:10.1186/s12905-026-04645-2)
Supplement: Supplementary file 1 [file 12905_2026_4645_MOESM12_ESM.pdf]

NIHR | National Institute for Health and Care Research

PROSPERO
International prospective register of systematic reviews

Ethanol sclerotherapy versus cystectomy in the management of endometrioma: A systematic review and meta-analysis

Sarah Bennet, Thomas Bainton, Jeffery Ahmed, Mark Johnson, Victoria Male, Sangeetha Nagenthiran

Review methods were amended after registration. Please see the revision notes and previous versions for detail.

Citation
Sarah Bennet, Thomas Bainton, Jeffery Ahmed, Mark Johnson, Victoria Male, Sangeetha Nagenthiran. Ethanol sclerotherapy versus cystectomy in the management of endometrioma: A systematic review and meta-analysis. PROSPERO 2024 CRD42024625920. Available from https://www.crd.york.ac.uk/PROSPERO/view/CRD42024625920.

REVIEW TITLE AND BASIC DETAILS

Review title

Ethanol sclerotherapy versus cystectomy in the management of endometrioma: A systematic review and meta-analysis

Review objectives

How does ethanol sclerotherapy differ to cystectomy in the management of endometrioma as assessed by recurrence rates, complication rates, symptoms, measures of ovarian reserve and fertility outcome?

SEARCHING AND SCREENING

Searches

EMBASE, MEDLINE, PMC, The Cochrane Library, Scopus

Restrictions: Unable to get full text, non-original research, case reports

Abstract only - authors contacted to request full data

Study design

Include: Randomised controlled trials, cohort, case-controls

Exclude: Non original research, case reports, case series, animal studies

ELIGIBILITY CRITERIA

Condition or domain being studied

Endometriosis is an inflammatory condition characterised by the presence of endometrial tissue outside the uterine cavity. Ovarian cysts can occur secondary to endometriosis (endometrioma), with menstrual-like fluid accumulating within the ovary, these can cause pain and are linked to subfertility. Importantly they are rarely seen in isolation being strongly associated with the presence of deep infiltrating endometriosis.

The management of endometriomata provides a clinical dilemma in the need to balance pain symptoms with fertility concerns. Historically, cystectomy was considered first line management, however the detrimental effect it can have on ovarian reserve means there is a need to identify alternative options. This is of particular importance as endometriosis is most active in young women, many of whom are planning, or may wish to plan, a pregnancy. Ethanol sclerotherapy is an increasingly used technique, which involves draining the endometrioma and filling it with ethanol. This chemically ablates the lining of the cyst wall reducing the rates of recurrence seen with incision and drainage, while minimising damage to ovarian tissue. It can be performed under outpatient ultrasound guidance or at laparoscopy. Ethanol sclerotherapy appears to be safe and effective although there is a lack in experimental data to support this.

Population

Women with endometrioma of reproductive age

Intervention(s) or exposure(s)

Ethanol sclerotherapy for ovarian endometrioma (Ultrasound guided and laparoscopic)

Comparator(s) or control(s)

Cystectomy for ovarian endometrioma

OUTCOMES TO BE ANALYSED

Main outcomes

Change in markers of ovarian reserve (AMH, AFC) from baseline to post procedure follow up

Measures of effect

Mean difference

Additional outcomes 1 change

Cyst recurrence - Odds ratio

Symptoms/QoL - narrative (EHP30 not used in any studies)

Pregnancy outcome (Pregnancy rate, live birth rate, oocytes retrieved, mature oocytes) - narrative/Odds ratio/Mean difference/Mean difference

Complications - Rate

DATA COLLECTION PROCESS

Data extraction (selection and coding)

Two authors will independently review titles and discard those that do not fit inclusion criteria. The abstracts of the remaining articles will then be reviewed and full page text assessed of all eligible studies to ensure they fit criteria. Any disagreements will be discussed with a third reviewer and and a consensus reached.
Data extracted will include study design, date of publication, sample size, baseline characteristics of participants, site of endometriosis, details of intervention, details of controls, follow up period, outcomes measured, study funding
Mean and standard deviations will be extracted for continuous data
2x2 tables for dichotomous data

Risk of bias (quality) assessment

Risk of bias will be assessed using Cochrane risk of bias tool for RCTs (RoB2) and ROBINS-I tool for observational studies. Two authors will independently assess risk of bias, any disagreements will be discussed with a third reviewer and a consensus reached.

PLANNED DATA SYNTHESIS

Strategy for data synthesis

PRISMA model
Summary data will be extracted from each study, mean and standard deviation for continuous data, 2x2 tables constructed for dichotomous data
A summary statistic will then be calculated for each intervention and a combined intervention effect will be estimated.
Statistical heterogenecity will be assessed by I^2 test

REVIEW AFFILIATION, FUNDING AND PEER REVIEW

Review team members

Dr Sarah Bennet (review guarantor and contact) Chelsea and Westminster NHS Foundation Trust V Imperial College London. England.

No conflict of interest declared.

Dr Thomas Bainton. Chelsea and Westminster NHS Foundation Trust. England.

No conflict of interest declared.

Mr Jeffery Ahmed. Chelsea and Westminster Foundation Trust. England.

No conflict of interest declared.

Professor Mark Johnson. Imperial College London. England.

No conflict of interest declared.

Dr Victoria Male. Imperial College London. England.

No conflict of interest declared.

Dr Sangeetha Nagenthiran. Chelsea and Westminster NHS Trust. England.

No conflict of interest declared.

Named contact

Dr Sarah Bennet (s.bennet@nhs.net). Chelsea and Westminster NHS Foundation Trust \/ Imperial College London. England.

Review affiliation

Chelsea and Westminster NHS Foundation Trust / Imperial College London

Funding source

The primary authors are employees of Chelsea and Westminster Hospital NHS Foundation Trust with time allocated for undertaking research relevant to their role.

Potential sources of additional funding, if required: Chelsea Centre for Minimally Invasive Gynaecology (CCMIG), London. CW+ Charity (Chelsea and Westminster Hospital Charity), London<\/div>

TIMELINE OF THE REVIEW

Review timeline

Start date: 16 December 2024. End date: 01 April 2025

Date of first submission to PROSPERO

12 December 2024

Date of registration in PROSPERO

16 December 2024

CURRENT REVIEW STAGE

Publication of review results

The intention is to publish the review once completed.The review will be published in English

Stage of the review at this submission 1 change

Review stage	Started	Completed
Pilot work	✓	✓
Formal searching/study identification	✓	✓
Screening search results against inclusion criteria	✓	✓
Data extraction or receipt of IPD	✓	
Risk of bias/quality assessment	✓	✓
Data synthesis	✓	

Review status

The review is currently planned or ongoing.

ADDITIONAL INFORMATION

PROSPERO version history 1 change

- Version 6.0, published 28 Jan 2026
- Version 5.0, published 19 Jan 2026
- Version 4.0, published 19 Jan 2026
- Version 3.0, published 06 Oct 2025
- Version 2.0, published 06 Oct 2025
- Version 1.1, published 24 Feb 2025
- Version 1.0, published 16 Dec 2024

Review conflict of interest
None known

Country
England

Medical Subject Headings
Cystectomy; Endometriosis; Female; Fertility; Humans; Ovarian Reserve; Sclerotherapy

Revision note 1 change
Outcome measures updated for symptom/QoL (plan to use EHP 30 but various other scoring sysytems described in literature) and pregnancy rate (initial plan for odds ration however not appropriate given available data)

Disclaimer
The content of this record displays the information provided by the review team. PROSPERO does not peer review registration records or endorse their content.

PROSPERO accepts and posts the information provided in good faith; responsibility for record content rests with the review team. The guarantor for this record has affirmed that the information provided is truthful and that they understand that deliberate provision of inaccurate information may be construed as scientific misconduct.

PROSPERO does not accept any liability for the content provided in this record or for its use. Readers use the information provided in this record at their own risk.

Any enquiries about the record should be referred to the named review contact
