# Supplementary material for: Ethanol sclerotherapy versus cystectomy in the management of endometriomata: a systematic review and meta-analysis
Source: BMC Womens Health. 2026 Jul 18;26:518. doi: 10.1186/s12905-026-04645-2 (PMC13663109; doi:10.1186/s12905-026-04645-2)
Supplement: Supplementary file 2 [file 12905_2026_4645_MOESM2_ESM.pdf]

Baseline AMH: Sclerotherapy vs Cystectomy

Effect size of each study
Estimated overall effect size
Estimated overall confidence interval
Confidence interval of effect size
No-effect value

Months	Study	Mean difference	MeanAMHsclerotherapy	SDsclerotherapy	MeanAMHcystectomy	SDcystectomy	Bias	n
1	Wang 2025	0.05	3.74	2.01	3.69	1.73	Critical	207
	Subgroup Overall	0.05						
3	Antonaci 2022	0.07	3.21	2.43	3.14	2.45	Serious	49
	Vaduva 2023 (20-29)	-0.20	3.24	2.26	3.44	3.10	Serious	20
	Vaduva 2023 (30-35)	-0.05	2.26	1.78	2.31	1.76	Serious	76
	Yu 2025	-0.11	1.34	.79	1.45	.61	Critical	67
	Popov 2019	0.30	3.40	.70	3.10	.50	High	34
	Tehrani 2022	-0.36	2.12	1.05	2.48	1.34	Some concerns	70
	Subgroup Overall	-0.02						
6	Garcia-Tejedor 2020	0.64	2.39	2.04	1.75	2.16	Critical	24
	Koo 2020	-0.37	2.90	3.35	3.27	3.20	Serious	71
	Antonaci 2022	0.07	3.21	2.43	3.14	2.45	Serious	49
	Mohtashami 2024	-0.32	1.14	1.53	1.46	1.07	Serious	88
	Sukur 2024	0.70	2.60	1.50	1.90	.90	Critical	51
	Subgroup Overall	0.14						
Overall		0.02						

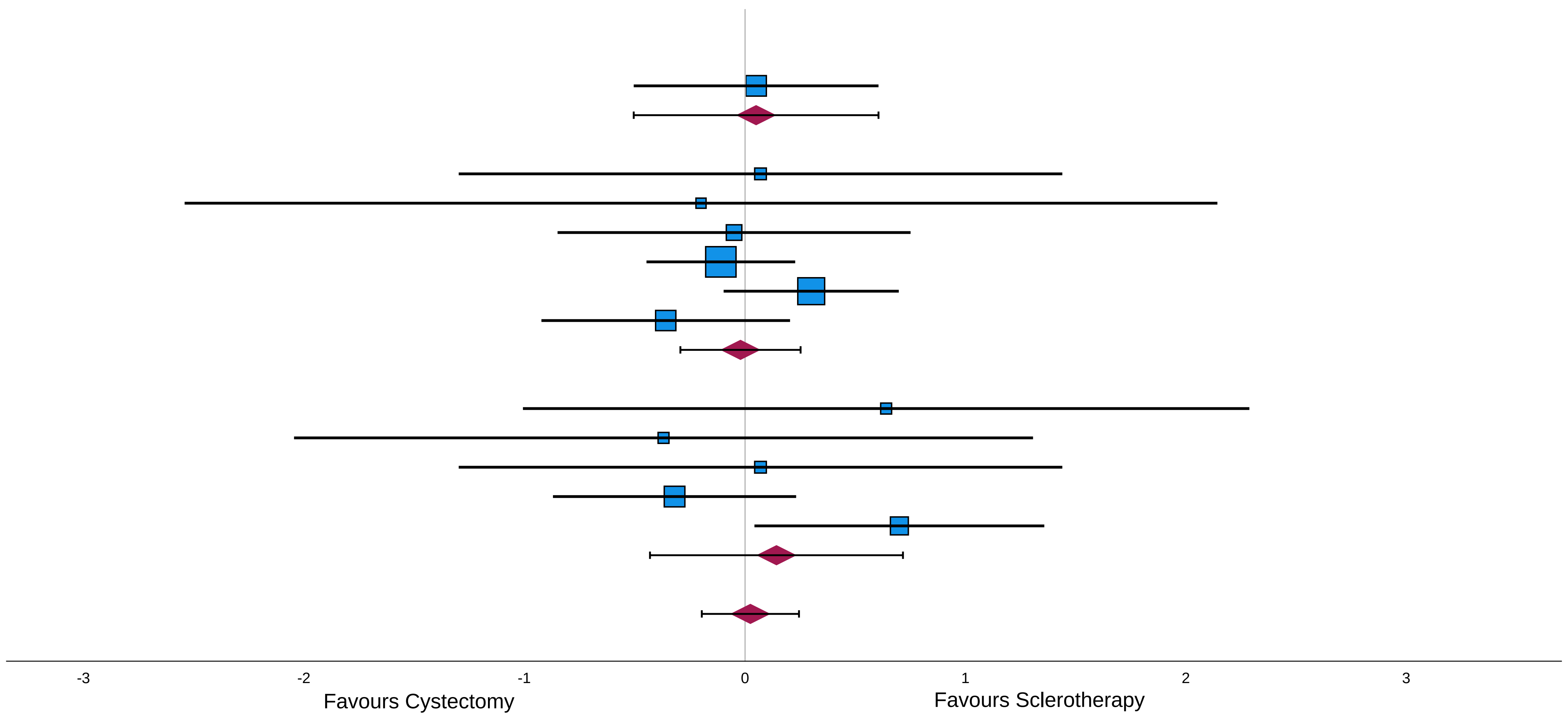

Model: Random-effects model

Heterogeneity: Tau-squared = 0.03, H-squared = 1.25, I-squared = 0.20

Test of between-subgroup homogeneity: Q = 0.27, df = 2, p-value = 0.87
