# Supplementary material for: Ethanol sclerotherapy versus cystectomy in the management of endometriomata: a systematic review and meta-analysis
Source: BMC Womens Health. 2026 Jul 18;26:518. doi: 10.1186/s12905-026-04645-2 (PMC13663109; doi:10.1186/s12905-026-04645-2)
Supplement: Supplementary file 3 [file 12905_2026_4645_MOESM3_ESM.pdf]

Post-procedure AMH: Sclerotherapy vs Cystectomy

- Effect size of each study
- Estimated overall effect size
- Estimated overall confidence interval
- Confidence interval of effect size
- No-effect value

Months	Study	Mean difference	MeanAMHsclerotherapy	SDsclerotherapy	MeanAMHcystectomy	SDcystectomy	Bias	n
1	Wang 2025	0.88	3.23	1.83	2.35	1.87	Critical	207
	Subgroup Overall	0.88						
3	Antonaci 2022	1.06	3.24	2.45	2.18	2.10	Serious	49
	Vaduva 2023 (20-29)	1.23	3.05	3.57	1.82	2.08	Serious	20
	Vaduva 2023 (30-35)	0.98	2.18	1.69	1.20	1.48	Serious	76
	Yu 2025	0.00	1.19	.98	1.19	.70	Critical	67
	Popov 2019	1.10	2.90	.40	1.80	.24	High	34
	Tehrani 2022	0.47	2.09	1.01	1.62	1.22	Some concerns	70
	Subgroup Overall	0.69						
6	Garcia-Tejedor 2020	0.83	2.02	1.88	1.19	.88	Critical	24
	Koo 2020	0.97	2.80	3.19	1.83	1.91	Serious	71
	Antonaci 2022	0.95	3.17	2.15	2.22	1.97	Serious	49
	Mohtashami 2024	0.13	1.00	.99	.87	1.23	Serious	88
	Sukur 2024	1.40	2.30	1.50	.90	.60	Critical	51
	Subgroup Overall	0.81						
Overall		0.75						

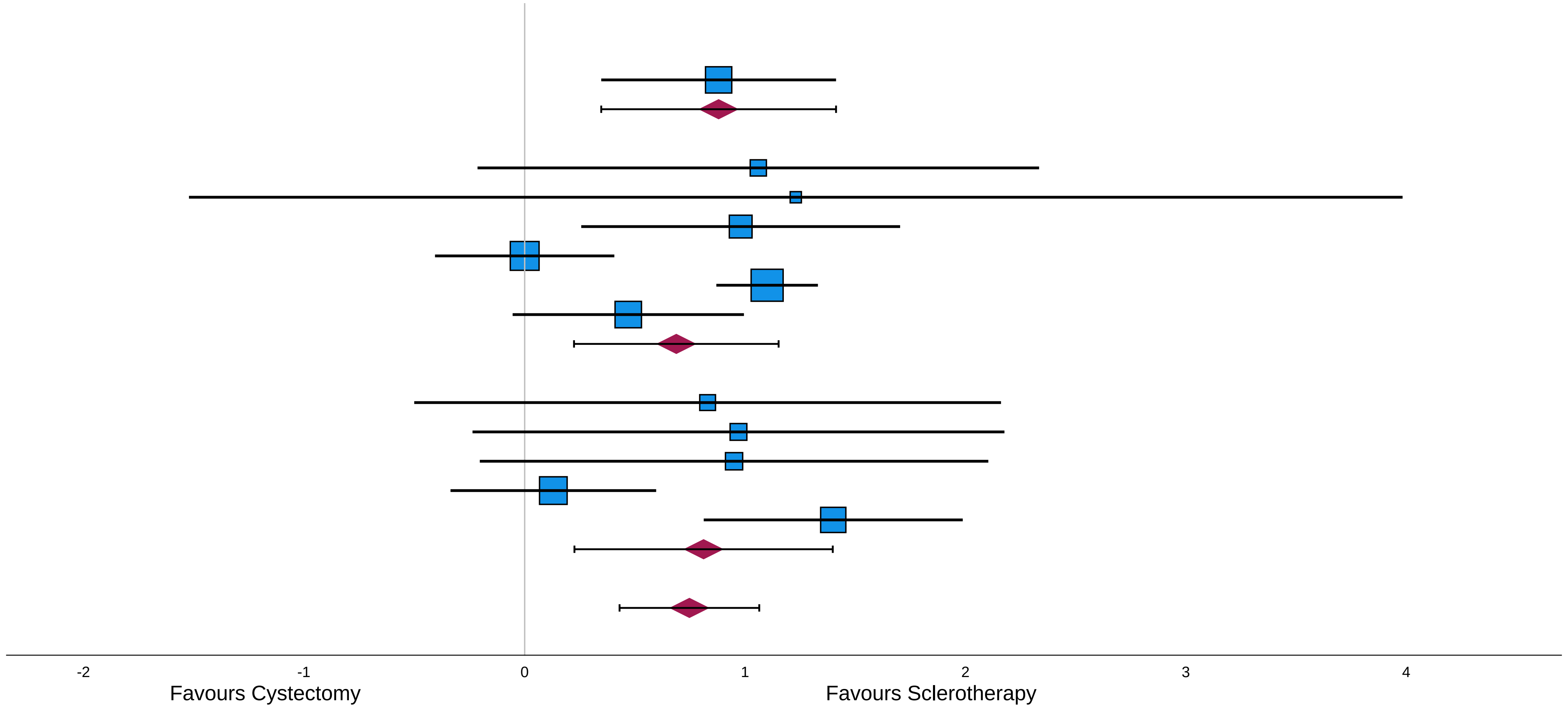

Model: Random-effects model

Heterogeneity: Tau-squared = 0.16, H-squared = 2.95, I-squared = 0.66

Test of between-subgroup homogeneity: Q = 0.30, df = 2, p-value = 0.86
