# Supplementary material for: Ethanol sclerotherapy versus cystectomy in the management of endometriomata: a systematic review and meta-analysis
Source: BMC Womens Health. 2026 Jul 18;26:518. doi: 10.1186/s12905-026-04645-2 (PMC13663109; doi:10.1186/s12905-026-04645-2)
Supplement: Supplementary file 4 [file 12905_2026_4645_MOESM4_ESM.pdf]

Sclerotherapy arm: Baseline AMH vs Post-procedure AMH

■ Effect size of each study
◆ Estimated overall effect size
Estimated overall confidence interval
| Confidence interval of effect size
— No-effect value

Months	Study	Mean difference	MeanAMHbaseline	SDbaseline	MeanAMHpost	SDpost	Bias	n
1	Wang 2025	-0.51	3.74	2.01	3.23	1.83	Critical	207
	Subgroup Overall	-0.51						
3	Antonaci 2022	0.03	3.21	2.43	3.24	2.45	Serious	49
	Vaduva 2023 (20-29)	-0.19	3.24	2.26	3.05	3.57	Serious	20
	Vaduva 2023 (30-35)	-0.08	2.26	1.78	2.18	1.69	Serious	76
	Yu 2025	-0.15	1.34	.79	1.19	.98	Critical	67
	Popov 2019	-0.50	3.40	.70	2.90	.40	High	34
	Tehrani 2022	-0.03	2.12	1.05	2.09	1.01	Some concerns	70
	Subgroup Overall	-0.23						
6	Garcia-Tejedor 2020	-0.37	2.39	2.04	2.02	1.88	Critical	24
	Koo 2020	-0.10	2.90	3.35	2.80	3.19	Serious	71
	Antonaci 2022	-0.04	3.21	2.43	3.17	2.15	Serious	49
	Mohtashami 2024	-0.14	1.14	1.53	1.00	.99	Serious	88
	Sukur 2024	-0.30	2.60	1.50	2.30	1.50	Critical	51
	Subgroup Overall	-0.18						
Overall		-0.27						

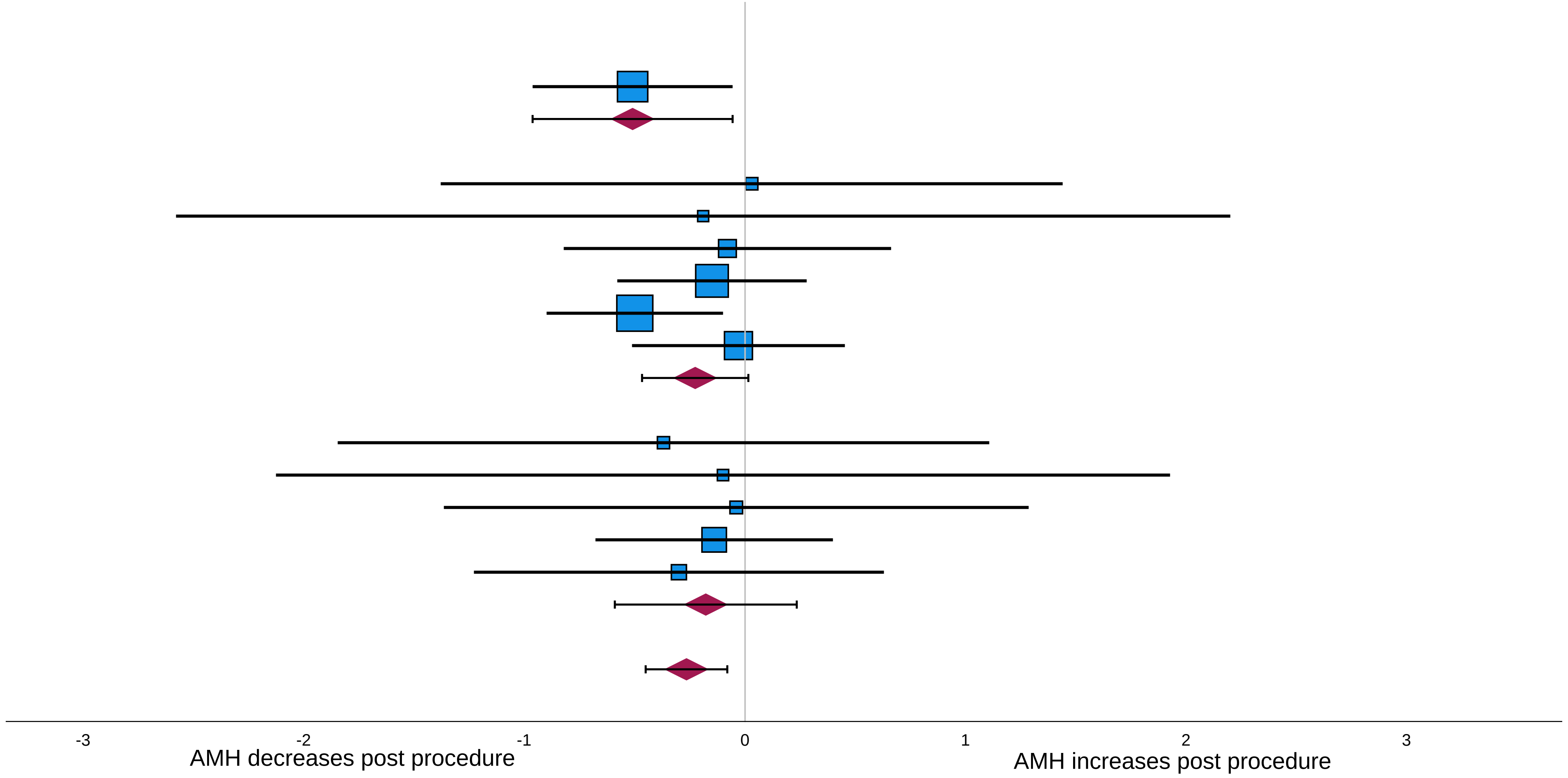

Model: Random-effects model

Heterogeneity: Tau-squared = 0.00, H-squared = 1.00, I-squared = 0.00

Test of between-subgroup homogeneity: Q = 1.39, df = 2, p-value = 0.50
