# Supplementary material for: Ethanol sclerotherapy versus cystectomy in the management of endometriomata: a systematic review and meta-analysis
Source: BMC Womens Health. 2026 Jul 18;26:518. doi: 10.1186/s12905-026-04645-2 (PMC13663109; doi:10.1186/s12905-026-04645-2)
Supplement: Supplementary file 7 [file 12905_2026_4645_MOESM7_ESM.pdf]

Cystectomy arm: Baseline AMH vs Post-procedure AMH (studies at high/critical risk of bias removed)

Effect size of each study
Estimated overall effect size
Estimated overall confidence interval
Confidence interval of effect size
No-effect value

Months	Study	Mean difference	MeanAMHbaseline	SDbaseline	MeanAMHpost	SDpost	Bias	n
3	Antonaci 2022	-0.96	3.14	2.45	2.18	2.10	Serious	49
	Vaduva 2023 (20-29)	-1.62	3.44	3.10	1.82	2.08	Serious	20
	Vaduva 2023 (30-35)	-1.11	2.31	1.76	1.20	1.48	Serious	76
	Tehrani 2022	-0.86	2.48	1.34	1.62	1.22	Some concerns	70
	Subgroup Overall	-0.97						
6	Koo 2020	-1.44	3.27	3.20	1.83	1.91	Serious	71
	Antonaci 2022	-0.92	3.14	2.45	2.22	1.97	Serious	49
	Mohtashami 2024	-0.59	1.46	1.07	.87	1.23	Serious	88
	Subgroup Overall	-0.83						
Overall		-0.86						

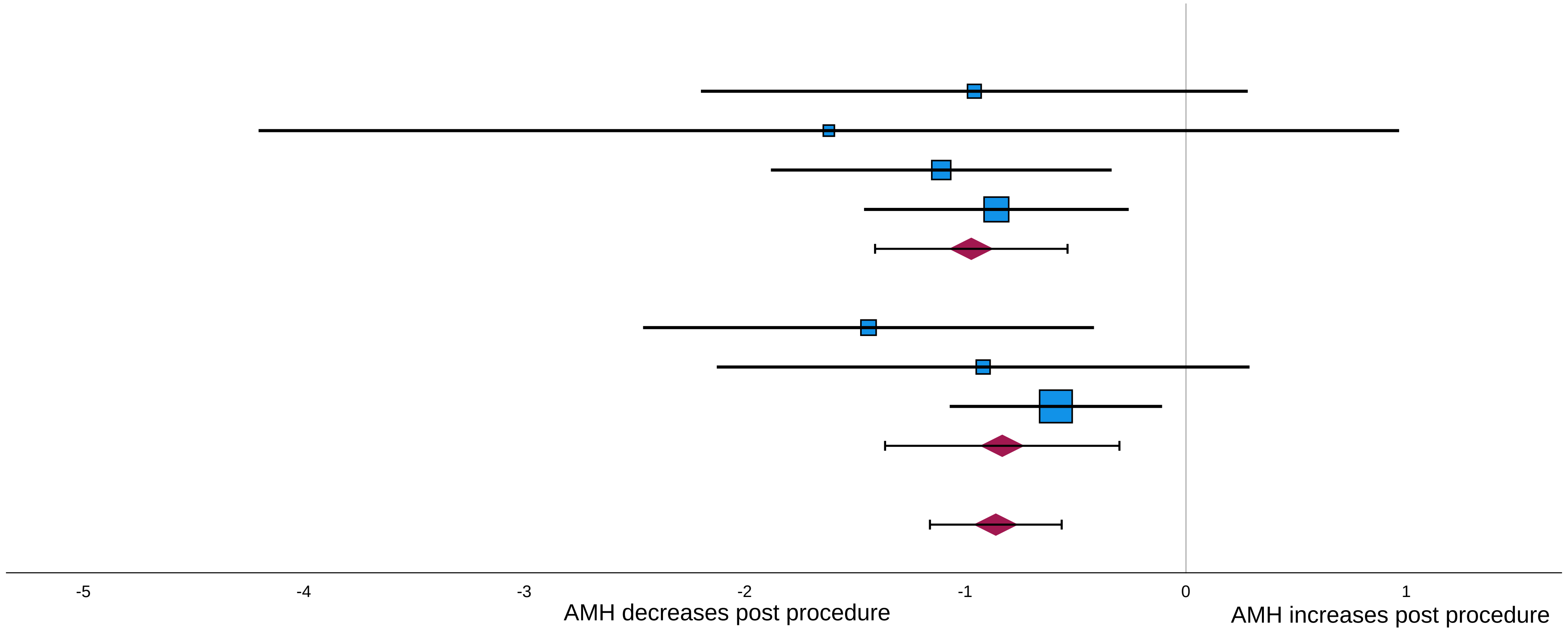

Model: Random-effects model

Heterogeneity: Tau-squared = 0.00, H-squared = 1.00, I-squared = 0.00

Test of between-subgroup homogeneity: Q = 0.16, df = 1, p-value = 0.69
