# Supplementary material for: Ethanol sclerotherapy versus cystectomy in the management of endometriomata: a systematic review and meta-analysis
Source: BMC Womens Health. 2026 Jul 18;26:518. doi: 10.1186/s12905-026-04645-2 (PMC13663109; doi:10.1186/s12905-026-04645-2)
Supplement: Supplementary file 8 [file 12905_2026_4645_MOESM9_ESM.pdf]

Effect size of each study
Estimated overall effect size
Estimated overall confidence interval
Confidence interval of effect size
No-effect value

Recurrence

Studydesign	Study	LogOR	Lower	Upper	Bias	n
Cohort	Noma 2001	-1.47	-3.57	0.63	Serious	100
	Koo 2020	1.36	-1.61	4.32	Serious	71
	Garcia-Tejedor 2020	1.86	-0.47	4.19	Serious	31
	Alborzi 2021	-1.15	-2.13	-0.18	Serious	101
	Antonaci 2022	1.95	-1.07	4.96	Serious	49
	Mohtashami 2024	0.39	-0.84	1.62	Serious	88
	Sukur 2024	-0.43	-4.39	3.53	Critical	51
	Wang 2025	1.56	0.60	2.52	Critical	207
	Subgroup Overall	0.41	-0.63	1.44		
RCT	Popov 2019	0.00	-3.97	3.97	High	36
	Tehrani 2022	-0.23	-1.17	0.71	Some concerns	70
	Subgroup Overall	-0.22	-1.13	0.70		
Overall		0.28	-0.57	1.12		

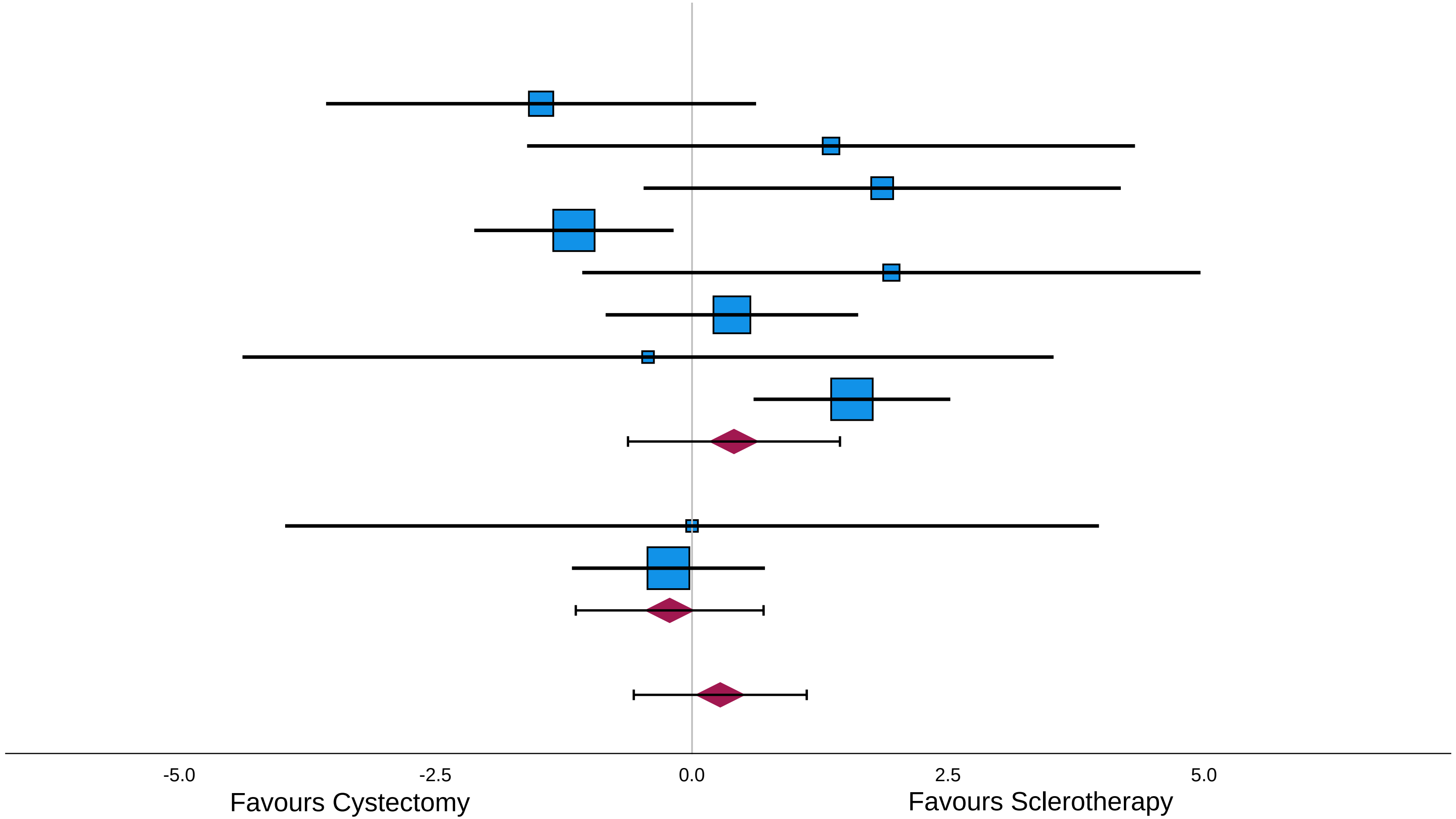

Model: Random-effects model

Heterogeneity: Tau-squared = 0.89, H-squared = 2.44, I-squared = 0.59

Test of between-subgroup homogeneity: Q = 0.79, df = 1, p-value = 0.37
