# Supplementary material for: Ethanol sclerotherapy versus cystectomy in the management of endometriomata: a systematic review and meta-analysis
Source: BMC Womens Health. 2026 Jul 18;26:518. doi: 10.1186/s12905-026-04645-2 (PMC13663109; doi:10.1186/s12905-026-04645-2)
Supplement: Supplementary file 9 [file 12905_2026_4645_MOESM10_ESM.docx]

# Additional File 10 (Pregnancy Outcomes)

| **Study ID** | **Method of conception** | **Interval between intervention and IVF** | **Mean number oocytes** | | **Mean mature oocytes** | | **Definition of Live Birth rate** | **Live birth events** | |  |
| --- | --- | --- | --- | --- | --- | --- | --- | --- | --- | --- |
|  |  |  | **Sclerotherapy mean±SD (n)** | **Cystectomy mean±SD (n)** | **Sclerotherapy mean±SD (n)** | **Cystectomy mean±SD (n)** |  | **Sclerotherapy**  **n (%)** | **Cystectomy**  **n (%)** | **p value** |
| **Takuma 2002** | IVF | 1 year (possibly earlier in cases of bilateral tubal obstruction) | 8.8±4.6 (10) | 7.3±4.9 (36) | N/A | N/A | N/A | N/A | N/A | N/A |
| **Yazbeck 2009** | IVF | At least 2 weeks | 11.4±6.1 (31) | 7.0±4.7 (26) | 10.4±5.4 (31) | 6.1±3.8 (26) | N/A | N/A | N/A | N/A |
| **Lee 2014** | IVF | ≤1 year for sclerotherapy  ≤ 5 years for cystectomy | 12.4±7.6 (29) | 8.2±4.7 (36) | 10.5±6.4 (29) | 6.9±3.7 (36) | Not specified | 11 (37.9%) | 12 (50.0%) | NS |
| **Alborzi 2021** | IVF | at time of ovum pickup for sclerotherapy  > 1 year for cystectomy | 7.95±5.18 (44) | 6.11±5.11 (57) | 6.66±4.69 (44) | 5.77±4.03 (57) | Not specified | 13 (29.5%) | 22 (38.6%) | 0.34 |
| **Antonaci 2022** | IVF | not reported for cystectomy,  2 weeks after sclerotherapy, | N/A | N/A | 6.82±4.23 (23) | 6.24±4.58 (26) | >22 weeks, per IVF cycle | 19 (36.5%) | 8 (14.3%) | 0.04 |
| **Sukur 2024** | IVF | ≤ 3 months for sclerotherapy,  ≤ 12 months for cystectomy, | 12.6±4.6 (20) | 5.1±3.6 (31) | 10.1±3.9 (20) | 4.2±3.5 (31) | >22 weeks, per IVF cycle | 13 (65.0%) | 9 (29.0%) | 0.017 |
| **Wang 2025 (LST)** | IVF | 4 weeks for both sclerotherapy and cystectomy | 11.8±6.2 (47) | 7.8±5.4 (69) | 6.4±6.8 (47) | 6.7±4.6 (69) | N/A | N/A | N/A | N/A |
| **Wang 2025 (UST)** | IVF | 4 weeks for both sclerotherapy and cystectomy | 11.2±6.9 (91) |  | 9.4±7.6 (7.6) |  | N/A | N/A |  | N/A |

**Table A10**: Extracted data on pregnancy outcomes: Oocytes retrieved, Mature oocytes and Live birth rate
