# Supplementary material for: Ethanol sclerotherapy versus cystectomy in the management of endometriomata: a systematic review and meta-analysis
Source: BMC Womens Health. 2026 Jul 18;26:518. doi: 10.1186/s12905-026-04645-2 (PMC13663109; doi:10.1186/s12905-026-04645-2)
Supplement: Supplementary file 11 [file 12905_2026_4645_MOESM1_ESM.docx]

# Additional File 1(Search terms)

Search strategy

**PubMed:**

P: ((endometrioma[MeSH Terms]) or (endometrio* or chocolate cyst or OMA))

I: ((sclerotherapy[MeSH Terms]) OR (sclerotherap* or sclerosing or CDS) OR (alcohol N5 inject*) OR (alcohol fixation) OR (ethanol))

C: ((ovarian cystectomy[MeSH Terms]) or (cystectom* or cyst removal or cyst stripping or cyst excision) or ((surgical management N5 cyst) or (cyst N3 removal) or (cyst N3 stripping) or (cyst N3 excision)) or (surgical excision or surgical resection or surgical stripping or surgical removal) or (laparoscopic excision or laparoscopic resection or laparoscopic stripping or laparoscopic removal or laparoscopic surgery))

**EMBASE:**

P: (endometrioma/ or endometriosis/ or ovarian endometriosis/) or (endometrio* or chocolate cyst or OMA)

I: (endoscopic sclerotherapy/ or sclerotherapy) or (sclerotherap* or sclerosing or CDS) or (alcohol adj5 inject* or alcohol fixation) or (ethanol.mp)

C: (ovarian cystectomy/ or laparoscopic ovarian cystectomy) OR (cystectom* or cyst removal or cyst stripping or cyst excision) OR ((surgical management adj5 cyst) or (cyst adj3 removal) or (cyst adj3 stripping) or (cyst adj3 excision)) or (surgical excision or surgical resection or surgical stripping or surgical removal) or (laparoscopic excision or laparoscopic resection or laparoscopic stripping or laparoscopic removal or laparoscopic surgery)))

**MEDLINE:**

P: (endometriosis/) or (endometrio* or chocolate cyst or OMA)

I: (Sclerotherapy/ or sclerosing solutions/) OR (sclerotherap* or sclerosing or CDS) OR (alcohol adj5 inject* or alcohol fixation) OR (ethanol.mp)

C: (laparoscopy/) OR (cystectom* or cyst removal or cyst stripping or cyst excision) OR ((surgical management adj5 cyst) or (cyst adj3 removal) or (cyst adj3 stripping) or (cyst adj3 excision)) or (surgical excision or surgical resection or surgical stripping or surgical removal) or (laparoscopic excision or laparoscopic resection or laparoscopic stripping or laparoscopic removal or laparoscopic surgery))

**SCOPUS**

P: (endometrio* or chocolate cyst or OMA)

I: (sclerotherap* or sclerosing or CDS) OR ( alcohol W5 inject* or alcohol fixation) OR (ethanol)

C: (cystectom* or cyst removal or cyst stripping or cyst excision) OR ((surgical management W5 cyst) or (cyst W3 removal) or (cyst W3 stripping) or (cyst W3 excision)) or (surgical excision or surgical resection or surgical stripping or surgical removal) or (laparoscopic excision or laparoscopic resection or laparoscopic stripping or laparoscopic removal or laparoscopic surgery)

**The Cochrane Library**

P: Endometrioma

I: Sclerotherapy

C: Ovarian cystectomy
