# Supplementary material for: Ethanol sclerotherapy versus cystectomy in the management of endometriomata: a systematic review and meta-analysis
Source: BMC Womens Health. 2026 Jul 18;26:518. doi: 10.1186/s12905-026-04645-2 (PMC13663109; doi:10.1186/s12905-026-04645-2)
Supplement: Supplementary file 12 [file 12905_2026_4645_MOESM8_ESM.docx]

# Additional File 8 (Recurrence)

| **Study** | **Sclerotherapy Recurrence events (%)** | **Cystectomy Recurrence events (%)** | **Follow up** | **Definition of recurrence** |
| --- | --- | --- | --- | --- |
| **Noma 2001** | 11 (14.9) | 1 (3.8) | at least 6 months, mean 26.2months | Not defined |
| **Garcia-Tejedor 2020** | 1 (5.9) | 4 (28.6) | from 6 months to up to 3yrs | presence of an adnexal cyst suggestive of endometrioma with a maximum diameter ≥30 mm in either ovary on ultrasound examination |
| **Alborzi 2021** | 15 (34.1) | 8 (14) | Up to 7 years | a persistent round-shaped, thick-walled cyst, filled with low echogenic fluid, more than 2 cm in diameter in the previous affected ovary |
| **Koo 2020** | 0(0) | 4 (7.8) | from 1 month, mean 23 months | Not defined |
| **Antonaci 2022** | 0(0) | 3 (11.5) | Up to 36 months | a cyst of any size |
| **Mohtashami 2024** | 5  (11.36) | 7 (15.9) | 2, 6, 12 months | 2cm not resolving on subsequent menstrual cycles |
| **Sukur 2024** | 0(0) | 0(0) | At time of IVF (within 3 months for sclerotherapy, 12 months for cystectomy) | Not defined |
| **Popov 2019** | 0(0) | 0(0) | Up to 16months | Not defined |
| **Tehrani 2022** | 17(48.58) | 15 (42.85) | 12 months | Not defined |
| **Wang 2025 (LST)** | 2 (4.3) | 14 (20.3) | 1 month | Not defined |
| **Wang 2025 (UST)** | 5(5.5) |  |  |  |

**Table A8: Table of data extracted on recurrence rates**
